# Lipoprotein Receptors Redundantly Participate in Entry of Hepatitis C Virus

**DOI:** 10.1371/journal.ppat.1005610

**Published:** 2016-05-06

**Authors:** Satomi Yamamoto, Takasuke Fukuhara, Chikako Ono, Kentaro Uemura, Yukako Kawachi, Mai Shiokawa, Hiroyuki Mori, Masami Wada, Ryoichi Shima, Toru Okamoto, Nobuhiko Hiraga, Ryosuke Suzuki, Kazuaki Chayama, Takaji Wakita, Yoshiharu Matsuura

**Affiliations:** 1 Department of Molecular Virology, Research Institute for Microbial Diseases, Osaka University, Osaka, Japan; 2 Department of Gastroenterology and Metabolism, Applied Life Sciences, Institute of Biomedical & Health Sciences, Hiroshima University, Hiroshima, Japan; 3 Department of Virology II, National Institute of Infectious Diseases, Tokyo, Japan; The Scripps Research Institute, UNITED STATES

## Abstract

Scavenger receptor class B type 1 (SR-B1) and low-density lipoprotein receptor (LDLR) are known to be involved in entry of hepatitis C virus (HCV), but their precise roles and their interplay are not fully understood. In this study, deficiency of both SR-B1 and LDLR in Huh7 cells was shown to impair the entry of HCV more strongly than deficiency of either SR-B1 or LDLR alone. In addition, exogenous expression of not only SR-B1 and LDLR but also very low-density lipoprotein receptor (VLDLR) rescued HCV entry in the SR-B1 and LDLR double-knockout cells, suggesting that VLDLR has similar roles in HCV entry. VLDLR is a lipoprotein receptor, but the level of its hepatic expression was lower than those of SR-B1 and LDLR. Moreover, expression of mutant lipoprotein receptors incapable of binding to or uptake of lipid resulted in no or slight enhancement of HCV entry in the double-knockout cells, suggesting that binding and/or uptake activities of lipid by lipoprotein receptors are essential for HCV entry. In addition, rescue of infectivity in the double-knockout cells by the expression of the lipoprotein receptors was not observed following infection with pseudotype particles bearing HCV envelope proteins produced in non-hepatic cells, suggesting that lipoproteins associated with HCV particles participate in the entry through their interaction with lipoprotein receptors. Buoyant density gradient analysis revealed that HCV utilizes these lipoprotein receptors in a manner dependent on the lipoproteins associated with HCV particles. Collectively, these results suggest that lipoprotein receptors redundantly participate in the entry of HCV.

## Author Summary

Hepatitis C virus (HCV) utilizes several receptors to enter hepatocytes, including scavenger receptor class B type 1 (SR-B1) receptor and low-density lipoprotein receptor (LDLR). HCV particles interact with lipoprotein and apolipoproteins to form complexes termed lipoviroparticles. Several reports have shown that SR-B1 and LDLR participate in the entry of lipoviroparticles through interaction with lipoproteins. However, the precise roles of SR-B1 and LDLR in HCV entry have not been fully clarified. In this study, we showed that SR-B1 and LDLR have a redundant role in HCV entry. In addition, we showed that very low-density lipoprotein receptor (VLDLR) played a role in HCV entry similar to the roles of SR-B1 and LDLR. Interestingly, VLDLR expression was low in the liver in contrast to the abundant expressions of SR-B1 and LDLR, but high in several extrahepatic tissues. Our data suggest that lipoprotein receptors participate in the entry of HCV particles associated with various lipoproteins.

## Introduction

More than 160 million individuals worldwide are infected with hepatitis C virus (HCV), which is especially troubling because HCV-induced cirrhosis and hepatocellular carcinoma are life-threatening diseases [1]. Current standard therapy combining peg-interferon (IFN), ribavirin (RBV) and a protease inhibitor has achieved a sustained virological response in over 80% of individuals infected with HCV genotype 1 [2]. In addition, many antiviral agents targeting non-structural proteins and host factors involved in HCV replication have been proven highly effective for chronic hepatitis C patients [3].

HCV belongs to the *Flaviviridae* family and possesses a single positive-stranded RNA genome with a nucleotide length of 9.6 kb. There are many reports on candidate molecules for the transportation of HCV into cells. CD81, which directly binds to HCV envelope glycoprotein E2, was first identified as an HCV receptor [4]. Scavenger receptor class B type 1 (SR-B1) was also identified as a co-receptor responsible for E2 binding to human hepatic cells by comparative binding studies [5]. Upon introduction of pseudotype particles bearing HCV envelope proteins (HCVpp) [6], claudin-1 (CLDN1) and occludin (OCLN) were identified as entry receptors for HCVpp into human kidney-derived HEK293 cells and mouse embryonic fibroblast-derived NIH3T3 cells, respectively [7, 8]. CD81, SR-B1, CLDN1 and OCLN are regarded as essential factors for HCV entry because mouse NIH3T3 cells and hamster CHO cells expressing these four factors permit entry of HCVpp [8]. In addition, development of a robust *in vitro* propagation system of HCV based on the genotype 2a JFH1 strain (HCVcc) has led to the identification of several entry factors, including epidermal growth factor receptor (EGFR) [9], Niemann-pick C1 Like 1 protein (NPC1L1) [10] and cell death-inducing DFFA-like effector B (CIDEB) [11].

Previous reports have shown that HCV particles derived from patient sera interact with lipoproteins and apolipoproteins to form complexes known as lipoviroparticles (LVPs) [12, 13]. The formation of LVPs is considered to have significant roles in HCV assembly and entry. Because several HCV receptor candidates are known to play crucial roles in lipid metabolism, these molecules are suggested to participate in HCV binding through interaction with virion-associated lipoproteins. SR-B1 is highly expressed in liver and acts as a binding receptor for mainly HDL to facilitate lipid uptake into hepatocytes. Low-density lipoprotein receptor (LDLR) is also a binding receptor for lipoproteins and widely expressed in various tissues including liver. However, the roles of SR-B1 and LDLR in HCV entry are not yet fully understood.

Recently, novel genome-editing techniques involving the use of zinc finger nucleases, transcription activator-like effector nucleases, and clustered regularly interspaced short palindromic repeats (CRISPR) and CRISPR-associated protein (CRISPR/Cas9) systems have been developed [14–16]. The CRISPR/Cas9 system is composed of guide RNA containing protospacer adjacent motif (PAM) sequences and Cas9 nuclease, which form RNA-protein complexes to cleave the target sequences; this system has already been used for the quick and easy establishment of gene-knockout mice and cancer cell lines [17, 18]. Because of the narrow host range and tissue tropism of HCV, robust *in vitro* HCV propagation is limited to the combination of HCVcc and human hepatoma-derived Huh7 cell clones. These novel genome-editing techniques have enabled the establishment of target gene-knockout Huh7 cells, which provide reliable tools to determine the precise roles of host factors in the lifecycle of HCV.

In this study, Huh7 cell lines deficient in both the SR-B1 and LDLR genes were established by using the CRISPR/Cas9 system and revealed that SR-B1 and LDLR redundantly participate in the entry of HCV. In addition, very low-density lipoprotein receptor (VLDLR), which is expressed highly in the peripheral tissues but only slightly in the liver and Huh7 cells, plays a role in HCV entry redundant to those played by SR-B1 and LDLR.

## Results

### SR-B1 is dispensable for HCV entry into Huh7 cells

**Fig 1:**
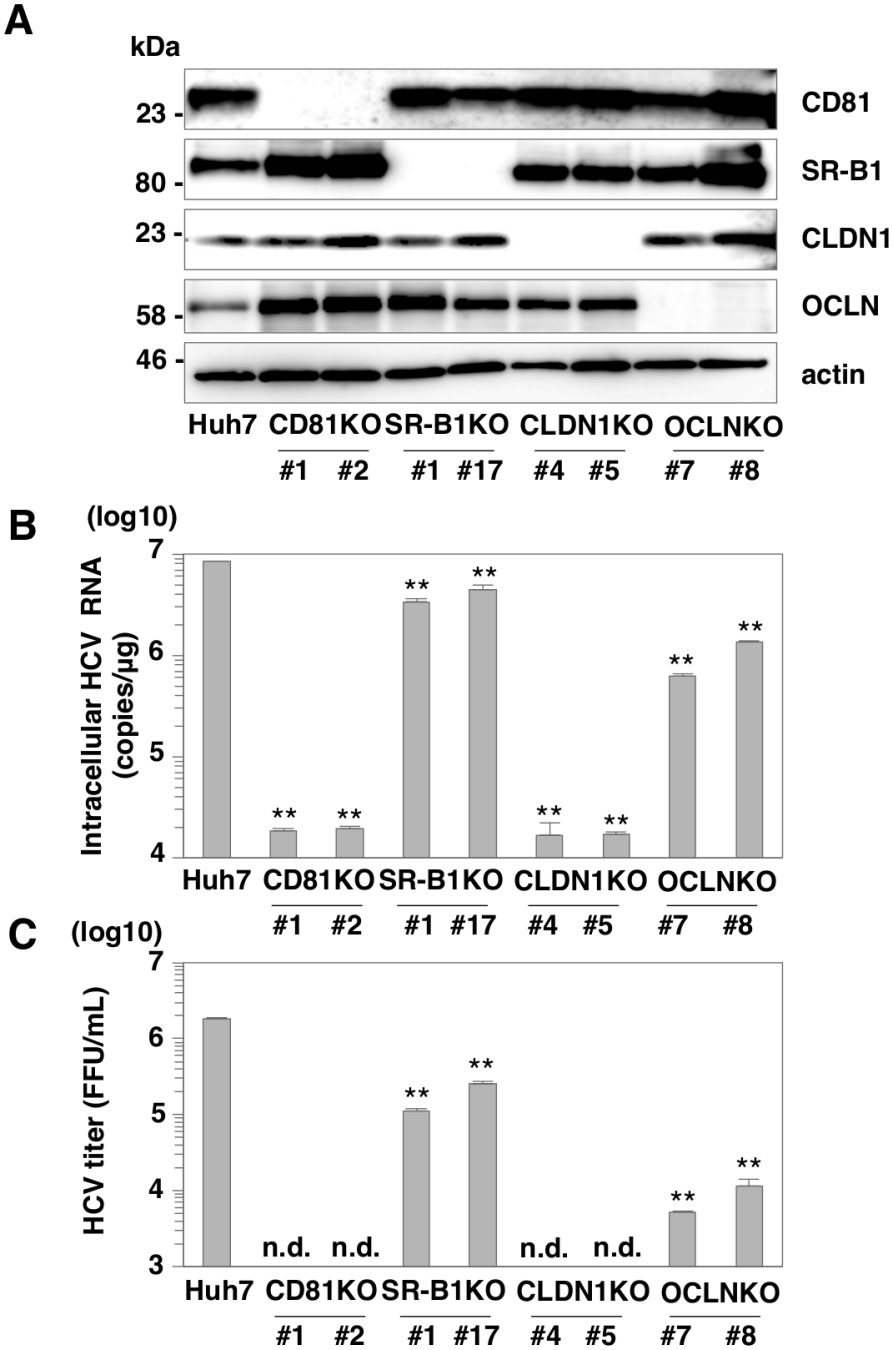
SR-B1 is dispensable for HCV entry into Huh7 cells. (A) Expressions of CD81, SR-B1, CLDN1, and OCLN in parental, CD81 KO, SR-B1 KO, CLDN1 KO and OCLN KO Huh7 cells were determined by immunoblotting analysis. (B) Cells were infected with HCVcc at an MOI of 1, and intracellular HCV RNA levels at 24 h post-infection were determined by qRT-PCR. (C) Infectious titers in the supernatants at 72 h post-infection were determined by focus-forming assay. In all cases, asterisks indicate significant differences (*P<0.05; **P<0.01) versus the results for parental Huh7 cells. “n.d.” indicates HCV titer below 10 FFU/ml.

Many receptor candidates and entry factors are known to be essential for HCV entry. Although previous reports have shown that CD81, SR-B1, CLDN1 and OCLN participate in HCV infection [8], the interplay among these molecules and precise roles in HCV entry are not fully understood. To clarify the involvement of these receptors in HCV entry in more detail, we used the CRISPR/Cas9 system to establish 2 clones for each of 4 knockout (KO) Huh7 cell lines respectively deficient in the CD81, SR-B1, CLDN-1 and OCLN genes (Fig 1A). Frame shift mutations in all alleles were confirmed by direct sequencing (S1A Fig). Cell viability was determined by the Cell Titer-Glo Luminescent Cell Viability Assay (S1B Fig, upper panel). Luciferase activities in these KO Huh7 cells are comparable to those of parental Huh7 cells. In addition, localization of lipid droplets which participate in lipid metabolism and in encapsidation of HCV was determined by the immunofluorescence assay (S1B Fig, middle panel). The mean numbers of lipid droplet per cell were determined by using ImageJ software (S1B Fig, lower panel). These KO and parental Huh7 cells exhibited similar localization and numbers of lipid droplets and morphologies. To examine the roles of these receptors in HCV entry, HCVcc was inoculated into these KO cells at a multiplicity of infection (MOI) of 1, and intracellular HCV RNA levels were determined by qRT-PCR at 24 h post-infection (Fig 1B). Huh7 cells deficient in either CD81, CLDN1 or OCLN exhibited a drastic reduction of the intracellular HCV RNA levels compared to those of parental Huh7 cells, in contrast to a slight reduction in those of SR-B1 (SR-KO) cells. In addition, infectious titers in the culture supernatants at 72 h post-infection exhibited little decrease in SR-KO cells, in contrast to the much greater decreases in CD81, CLDN1 or OCLN KO Huh7 cells (Fig 1C). To further confirm the effect of SR-B1 deficiency in HCV infection, SR-KO Huh7.5.1 cells were established (S2 Fig). The intracellular HCV RNA levels in SR-KO Huh7.5.1 cells at 24 h post-infection were slightly reduced compared to parental Huh7.5.1 cells, as seen in Huh7 cells. These results suggest that SR-B1 is dispensable for HCV entry into both Huh7 and Huh7.5.1 cells.

### SR-B1 and LDLR have a redundant role in HCV entry

**Fig 2:**
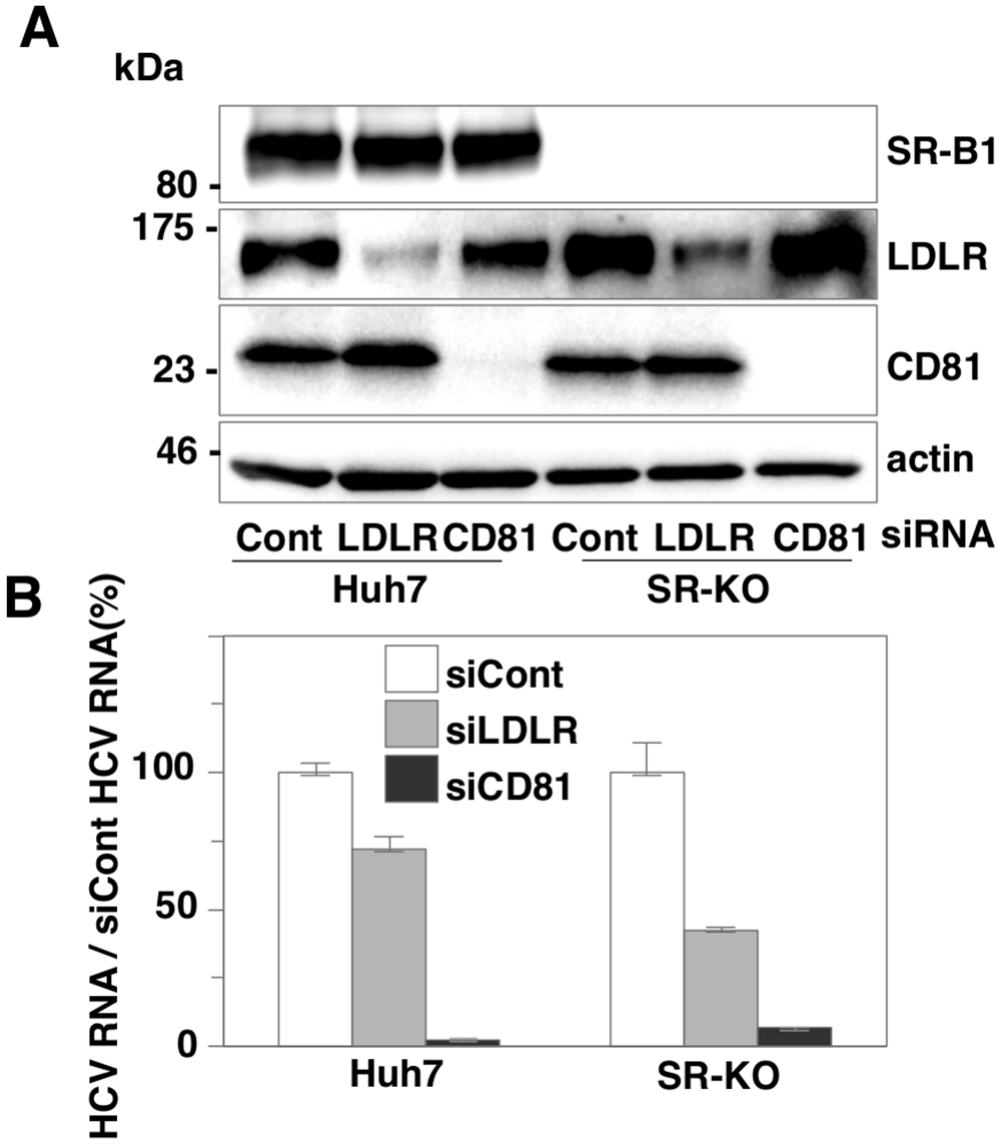
Deficiencies of SR-B1 and LDLR impair HCV entry. (A) Expressions of SR-B1, LDLR and CD81 in parental and SR-KO Huh7 cells transfected with siRNAs targeting LDLR or CD81 were determined by immunoblotting analysis at 48 h post-transfection. (B) Parental and SR-KO Huh7 cells were infected with HCVcc at an MOI of 1 at 48 h post-transfection with siRNAs targeting LDLR or CD81, and intracellular HCV RNA levels were determined at 24 h post-infection by qRT-PCR.

**Fig 3:**
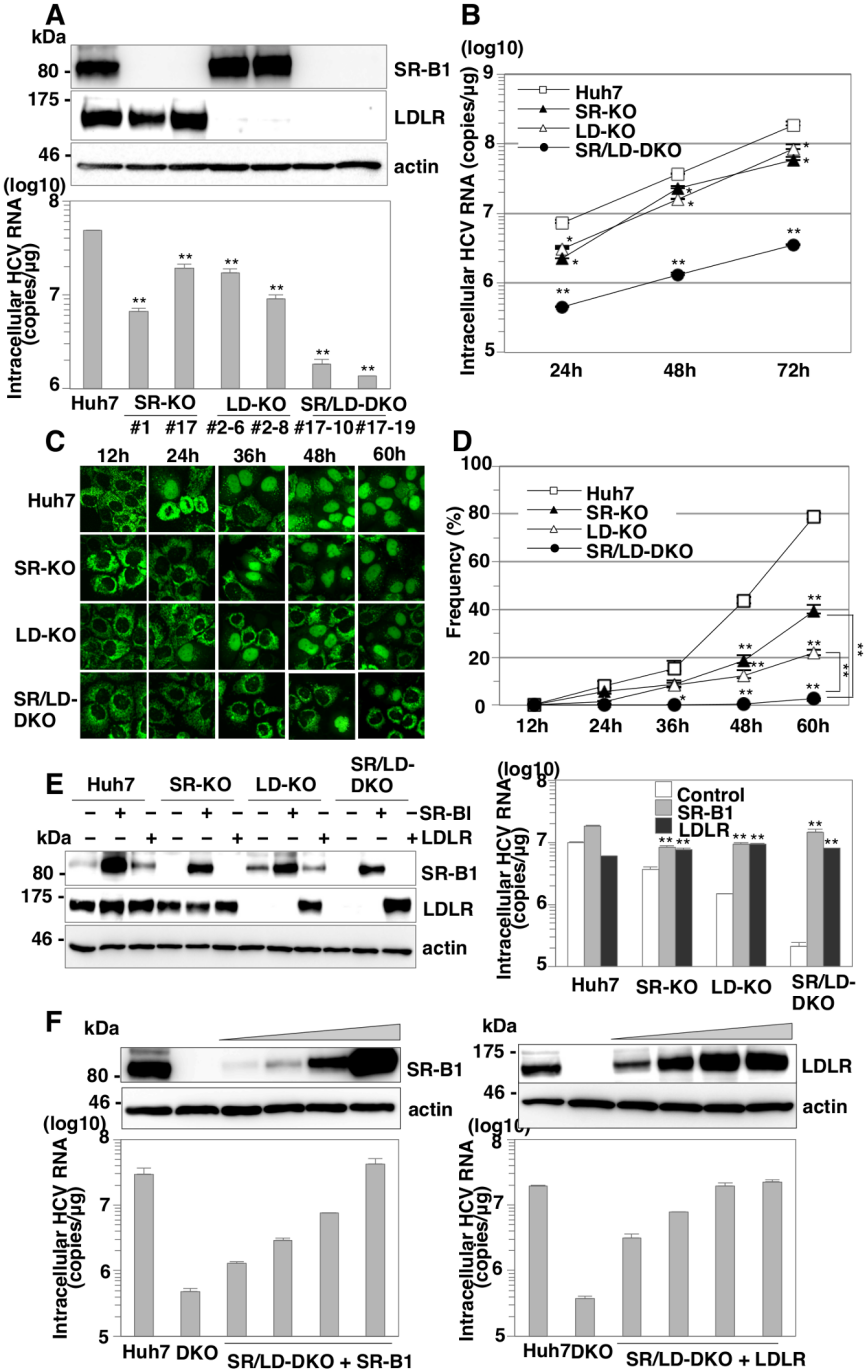
SR-B1 and LDLR have a redundant role in HCV entry. (A) Expressions of SR-B1 and LDLR in parental, SR-KO, LD-KO and SR/LD-DKO Huh7 cells were determined by immunoblotting analysis (upper panel). Cells were infected with HCVcc at an MOI of 1, and intracellular HCV RNA levels were determined at 24 h post-infection by qRT-PCR (lower panel). (B) Parental, SR-KO, LD-KO and SR/LD-DKO Huh7 cells were infected with HCVcc at an MOI of 1, and intracellular HCV RNA levels were determined at 24, 48 and 72 h post-infection by qRT-PCR. (C, D) Fluorescence localizations in parental, SR-KO, LD-KO and SR/LD-KO Huh7 cells were observed with a confocal microscope, upon infection with HCVcc at an MOI of 1 at 12, 24, 36, 48 and 60 h post-infection. The frequency is shown as the ratio of infected cells to total cells. (E) SR-B1 and LDLR were exogenously expressed in parental, SR-KO, LD-KO and SR/LD-DKO Huh7 cells by infection with lentiviral vectors. Expressions of SR-B1 and LDLR in these cells were determined by immunoblotting analysis (left panel). Cells were infected with HCVcc at an MOI of 1 and intracellular HCV RNA levels were determined at 24 h post-infection by qRT-PCR (right panel). (F) SR-B1 and LDLR were exogenously expressed in SR/LD-DKO Huh7 cells by infection with different amount of lentiviral vectors. Expressions of SR-B1 and LDLR in these cells were determined by immunoblotting analysis (upper panel). Cells were infected with HCVcc at an MOI of 1 and intracellular HCV RNA levels were determined at 24 h post-infection by qRT-PCR (lower panel). Asterisks indicate significant differences (*P<0.05; **P<0.01) versus the results for control cells.

Because both SR-B1 and LDLR have been reported to be entry factors for lipid-associated HCV particles, we hypothesized that LDLR can compensate for the role of SR-B1 in HCV entry. To examine the potential of a redundant role between SR-B1 and LDLR, the effect of siRNA-mediated knockdown of LDLR on HCV entry was examined in parental and SR-KO Huh7 cells. The efficiencies of siRNA-mediated knockdown were confirmed by immunoblotting (Fig 2A). Although intracellular viral RNA levels in cells infected with HCVcc were drastically reduced in both parental and SR-KO Huh7 cells by the knockdown of CD81, those in SR-KO cells were lower than those in parental cells by the knockdown of LDLR (Fig 2B). To further examine the role of SR-B1 and LDLR in HCV entry, 2 clones for LDLR KO (LD-KO) Huh7 cells and 2 clones for SR-B1 and LDLR double KO (SR/LD-DKO) Huh7 cells were established by the CRIPSR/Cas9 system (Fig 3A upper panel). Frame shift mutations in all alleles were confirmed by direct sequencing (S1A Fig). Intracellular viral RNA levels in SR/LD-DKO Huh7 cells infected with HCVcc at an MOI of 1 were about 30 times lower than those in parental Huh7 cells at 24 h post-infection, in contrast to the slight reduction of RNA replication in SR-KO and LD-KO cells (Fig 3A, lower panel). In addition, intracellular HCV RNA levels in SR/LD-DKO Huh7 cells were lower than those in SR-KO and LD-KO Huh7 cells at all time points after infection (Fig 3B). To visualize the dissemination of HCV infection, a fluorescence-based live cell reporter system was used [19]. Translocation of GFP from the cytoplasm to nucleus was observed in Huh7 cells stably expressing GFP-NLS-IPS upon infection with HCV through cleavage of the IPS-1 sequence by NS3-4A protease. Nuclear localization of GFP was observed from 24 h post-infection in parental, SR-KO, and LD-KO Huh7 cells upon infection with HCVcc at an MOI of 1, while it was detected from 48 h post-infection in SR/LD-DKO Huh7 cells (Fig 3C and 3D).

To further confirm the redundant role of SR-B1 and LDLR in HCV entry, the effects of exogenous expression of SR-B1 or LDLR in SR-KO, LD-KO and SR/LD-DKO Huh7 cells were examined (Fig 3E). Although HCV RNA levels in SR-KO, LD-KO and SR/LD-DKO Huh7 cells were lower than those in parental Huh7 cells at 24 h post-infection with HCVcc at an MOI of 1, exogenous expression of either SR-B1 or LDLR enhanced the RNA in SR-KO, LD-KO and SR/LD-DKO Huh7 cells to levels comparable to those in parental cells. In addition, HCV RNA levels were increased in accord with the expression levels of SR-B1 and LDLR (Fig 3F), suggesting that SR-B1 and LDLR redundantly participate in HCV entry.

To rule out the possibility that expression of SR-B1 and LDLR enhances HCV RNA replication, *in vitro*-transcribed subgenomic HCV RNA of the JFH1 strain was electroporated into SR-KO and LD-KO cells with or without expression of SR-B1 and LDLR and cultured in medium containing G418 for a month. Exogenous expression of SR-B1 and LDLR in each of the KO cells exhibited no significant effect on the colony formation of SGR cells (S3A Fig). Next, *in vitro*-transcribed Fluc RNA and subgenomic HCV RNA containing NanoLuc were electroporated into parental, SR-KO, LD-KO and SR/LD-DKO Huh7 cells and luciferase activities were determined at 24, 48, 72 h post-electroporation (S3B Fig). Luciferase activities were similar in parental and KO Huh7 cells, indicating that replication efficiencies of HCV RNA are comparable among these cells. To further examine the roles of SR-B1 and LDLR in HCV replication, replication of HCV RNA and expression of viral protein in each three clones derived from parental, SR-KO, LD-KO and SR/LD-DKO Huh7 cells were examined by qRT-PCR and immunoblotting, respectively (S3C Fig). HCV RNA (upper panel) and NS5A protein (lower panel) in these cells were comparable among all clones, suggesting that SR-B1 and LDLR are not involved in HCV replication. In addition, to examine the effect of SR-B1 and LDLR in particle production, *in vitro*-transcribed full-length HCV RNA was electroporated into SR/LD-DKO Huh7 cells expressing SR-B1 or LDLR, and infectious titer in the culture supernatants at early phase post-electroporation was determined by focus forming assay (S3D Fig). Deficiencies of SR-B1 and LDLR gene exhibited no significant effect on infectious titers in the supernatants, suggesting that neither SR-B1 nor LDLR is involved in particle production of HCV.

### VLDLR has a similar role with SR-B1 and LDLR in HCV entry

**Fig 4:**
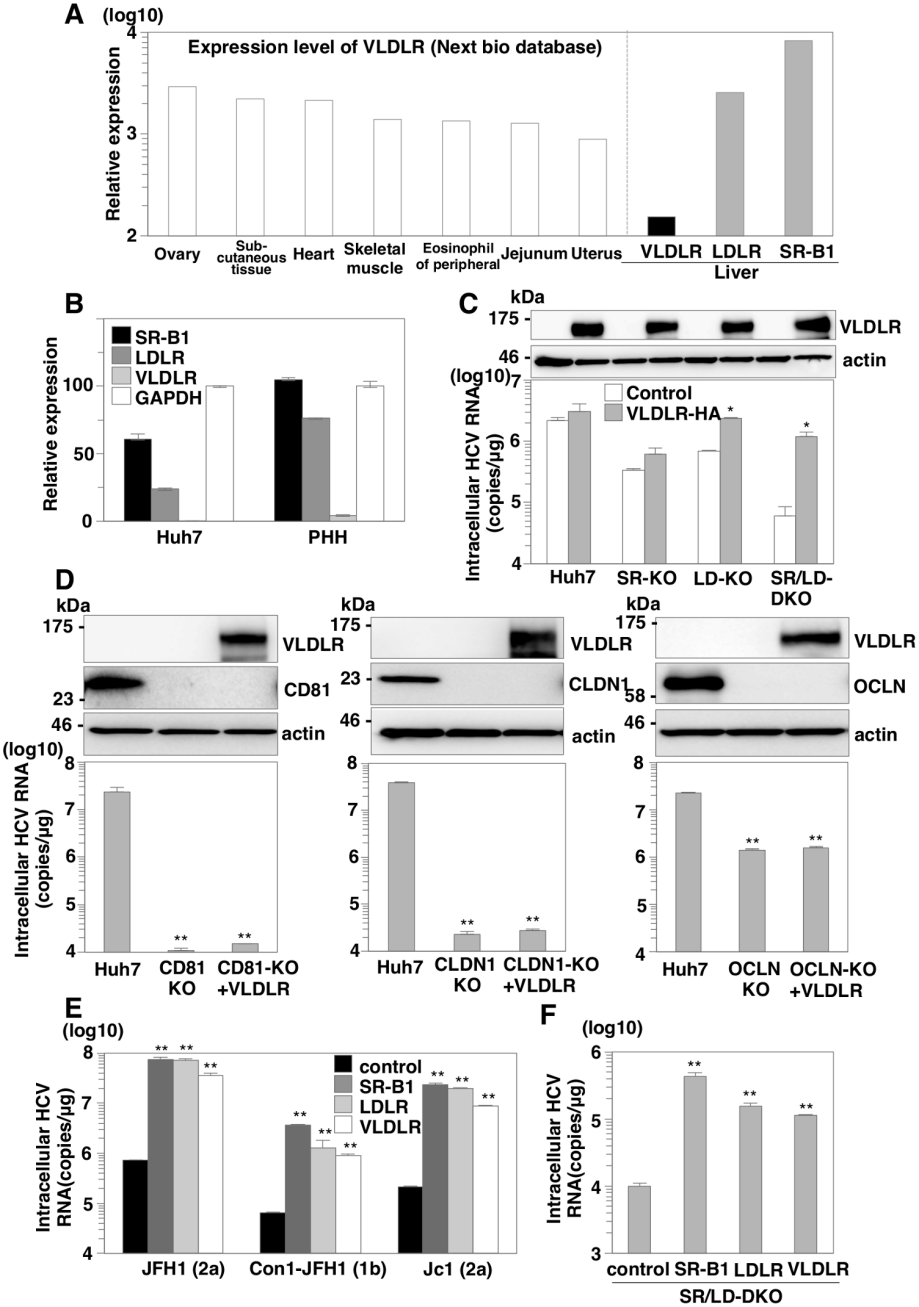
VLDLR has a similar role with SR-B1 and LDLR in HCV entry. (A) Relative mRNA expression of VLDLR in various tissues was determined using the NextBio Body Atlas application. (B) mRNA expression of SR-B1, LDLR, VLDLR and GAPDH in Huh7 cells and primary human hepatocyte (PHH) were determined by qRT-PCR. Relative expression levels of mRNA were calculated based on the expression level of GAPDH. (C) VLDLR-HA was exogenously expressed in parental, SR-KO, LD-KO and SR/LD-DKO Huh7 cells by infection with lentiviral vectors. Expressions of VLDLR in these cells were determined by immunoblotting analysis (upper panel). Parental, CD81 KO and SR/LD-DKO Huh7 cells expressing SR-B1, LDLR or VLDLR were infected with HCVcc at an MOI of 1 and intracellular HCV RNA levels were determined at 24 h post-infection (lower panel). (D) VLDLR-HA was exogenously expressed in CD81 KO, CLDN1 KO and OCLN KO Huh7 cells by infection with lentiviral vectors. Expressions of VLDLR, CD81, CLDN1 and OCLN in these cells were determined by immunoblotting analysis (upper panel). Cells were infected with HCVcc at an MOI of 1 and intracellular HCV RNA levels were determined at 24 h post-infection by qRT-PCR (lower panel). (E) SR-B1, LDLR and VLDLR were exogenously expressed in SR/LD-DKO Huh7 cells by infection with lentiviral vectors. Cells were infected with Con1-JFH1 or Jc1 at an MOI of 1, and intracellular HCV RNA levels were determined at 24 h post-infection by qRT-PCR. (F) Sera (100μl) from chimeric mice infected with HCV were inoculated into SR/LD-DKO Huh7 cells expressing either SR-B1, LDLR or VLDLR in 24 well plate. Intracellular HCV RNA levels were determined at 72 h post-infection. In all cases, asterisks indicate significant differences (*P<0.05; **P<0.01) versus the results for control cells.

A search using the web-based search engine NextBio (NextBio, Santa Clara, CA) revealed that VLDLR, a member of the LDLR family, is expressed at a high level in peripheral tissues, and at a low level in the liver (Fig 4A). Furthermore, expression levels of SR-B1 and LDLR are high in Huh7 cells and primary human hepatocytes (PHH), while those of VLDLR are quite low (Fig 4B). VLDLR belongs to the family of lipoprotein receptors and is structurally homologous to LDLR. Therefore, we considered that VLDLR may also be involved in HCV entry. To examine the role of VLDLR in HCV entry, VLDLR was expressed in SR-KO, LD-KO and SR/LD-DKO Huh7 cells by a lentiviral vector (Fig 4C, upper panel). HCVcc was inoculated into cells at an MOI of 1 and intracellular HCV RNA levels were determined by qRT-PCR at 24 h post-infection (Fig 4C, lower panel). Exogenous expression of VLDLR rescued HCV entry in SR/LD-DKO cells but not in parental Huh7 cells, suggesting that VLDLR expression can compensate for the roles of SR-B1 or LDLR in HCV entry. Very recently, Ujino et al. showed that VLDLR variant 2 participates in HCV entry independent from CD81-mediated HCV entry [20]. To further examine the role of VLDLR in HCV entry, VLDLR was expressed in CD81 KO, CLDN1 KO and OCLN KO Huh7 cells. In contrast to expression of VLDLR in SR/LD-DKO Huh7 cells, exogenous expression of VLDLR in CD81, CLDN1 and OCLN KO cells exhibited no effect on HCV entry upon infection with HCVcc (Fig 4D). Furthermore, HCVcc was inoculated into CD81 KO and SR/LD-DKO cells expressing either variant 1 or variant 2 of VLDLR at an MOI of 1 and intracellular HCV RNA levels and expression of NS5A were determined by qRT-PCR and immunofluorescense assay, respectively (S4 Fig). The levels of HCV RNA and NS5A expression in VLDLR expressing CD81 KO cells were comparable to that of control CD81 KO cells. These results suggest that the role of VLDLR cannot compensate the role of CD81, CLDN1 and OCLN. To examine the role of lipoprotein receptors in the entry of other genotypes of HCV, chimeric HCVcc of genotype 1b and 2a, the Con1-JFH1 and Jc1 viruses were used. Expression of SR-B1, LDLR or VLDLR enhanced the entry of Con1-JFH1 and Jc1 viruses in SR/LD-DKO Huh7 cells at 24 h post-infection (Fig 4E), as seen in JFH1 infection. To further examine the redundant role of lipoprotein receptors in the entry of HCV derived from *in vivo*, sera of mice with chimeric human livers infected with HCVcc were inoculated into SR/LD-DKO Huh7 cells expressing either SR-B1, LDLR or VLDLR, and the HCV RNA levels were determined by qRT-PCR (Fig 4F). Exogenous expression of SR-B1, LDLR or VLDLR recovered susceptibility of SR/LD-DKO Huh7 cells to the mice-derived HCV. These results suggest that VLDLR has a similar role with SR-B1 and LDLR in the entry of HCV derived from not only cell culture but also from *in vivo*.

### SR-B1, LDLR and VLDLR participate in the binding step of HCV entry

**Fig 5:**
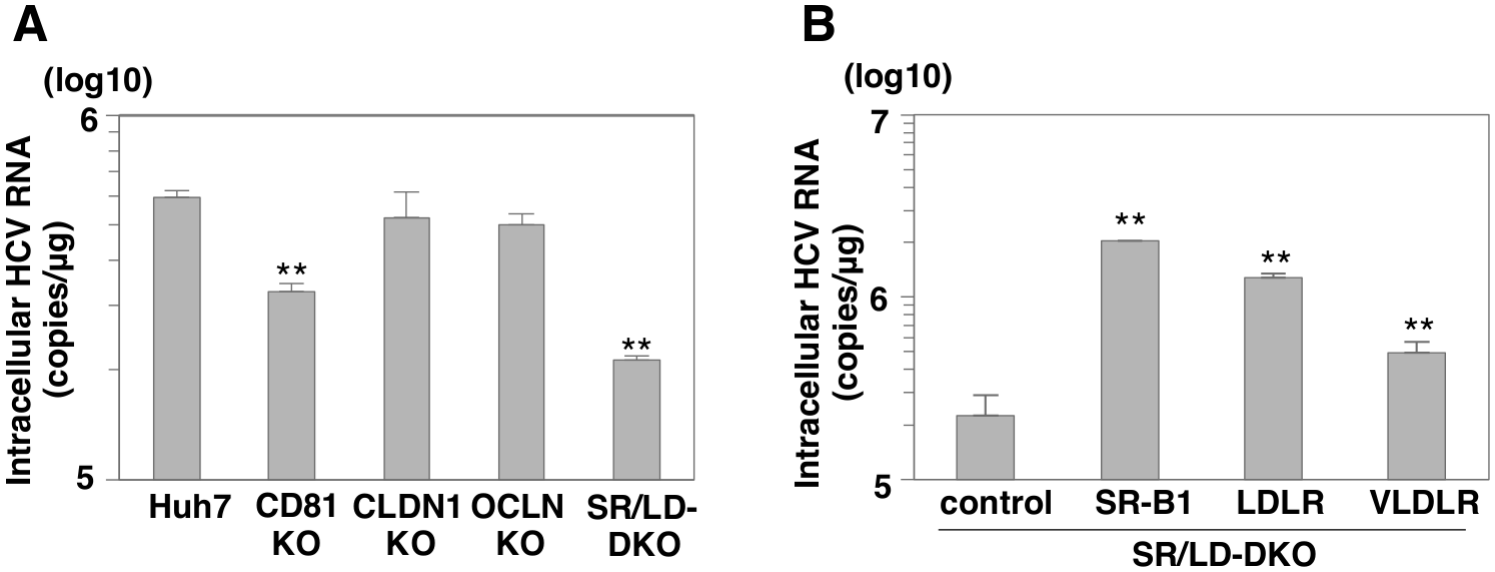
SR-B1, LDLR and VLDLR participate in the binding step of HCV entry. (A) Parental, CD81 KO, CLDN KO, OCLN KO and SR/LD-DKO Huh7 cells were inoculated with HCVcc at an MOI of 1 at 4°C for 1 h and washed three times with PBS, and HCV RNA levels were determined after the binding. (B) SR/LD-DKO Huh7 cells expressing SR-B1, LDLR or VLDLR were infected with HCVcc at an MOI of 1 at 4°C for 1 h and washed three times with PBS, and HCV RNA levels were determined after the binding. In all cases, asterisks indicate significant differences (*P<0.05; **P<0.01) versus the results for control cells.

To determine the roles of lipoprotein receptors in HCV entry in greater detail, binding assay was performed in SR/LD-DKO Huh7 cells expressing SR-B1, LDLR or VLDLR. HCVcc were inoculated into cells, incubated at 4°C for 1 h and then washed three times with phosphate-buffered saline (PBS) to remove unbound particles. HCV RNA levels were determined by qRT-PCR immediately following binding (Fig 5A and 5B). The intracellular HCV RNA levels were significantly lower in CD81 KO and SR/LD-DKO Huh7 cells, but were comparable in CLDN1 KO and OCLN KO Huh7 cells in compared with parental Huh7 cells. In addition, exogenous expression of SR-B1, LDLR or VLDLR in SR/LD-DKO Huh7 cells rescued the binding step, suggesting that lipoprotein receptors participate in the binding step of HCV entry.

### Lipid binding and lipid uptake of lipoprotein receptors participate in HCV entry

**Fig 6:**
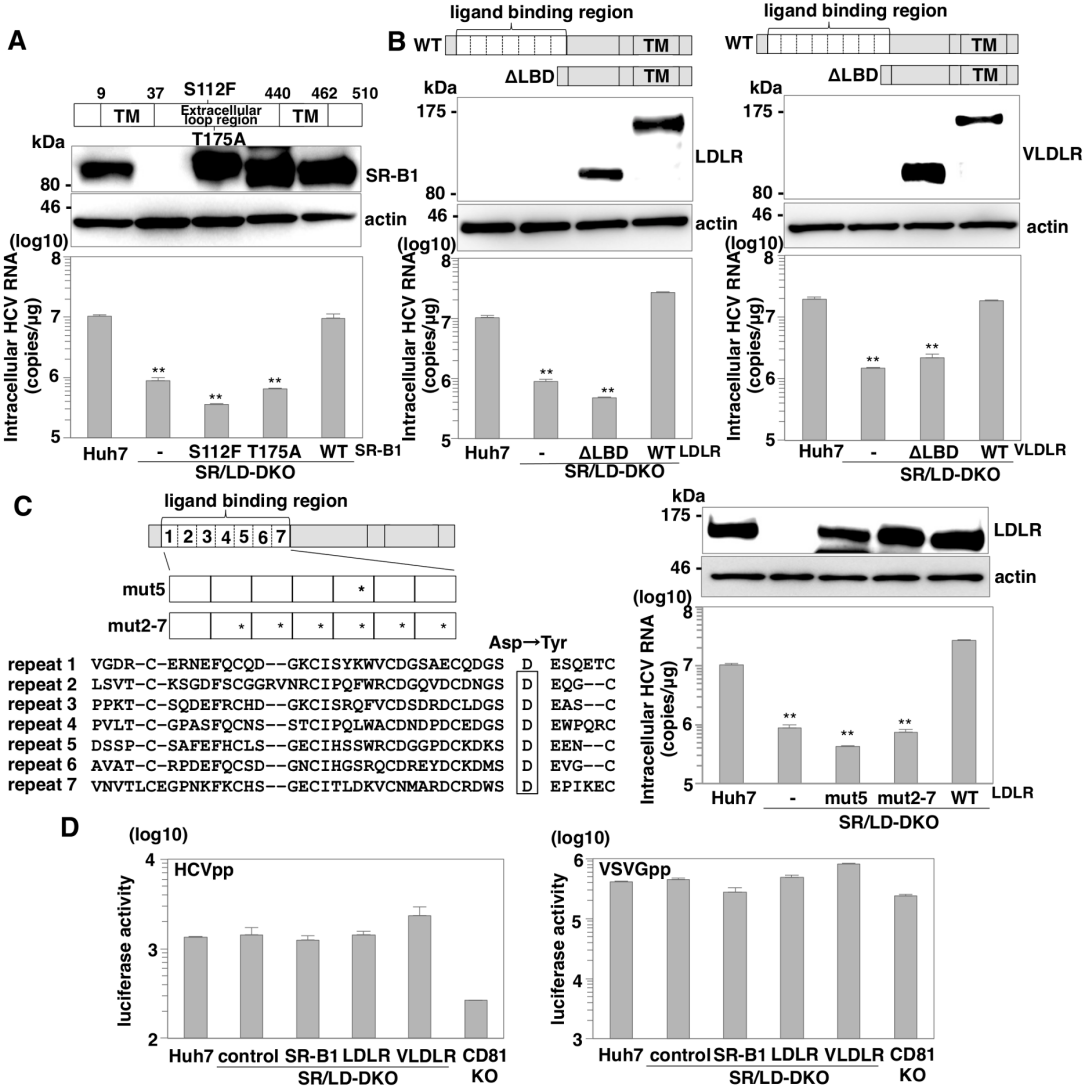
Lipid binding and lipid uptake of lipoprotein receptors participate in HCV entry. Schematics were shown for the SR-B1, LDLR and VLDLR mutants (upper panels of A and B, and left panel of C). (A) S112F- and T175A-SR-B1 were generated to examine the significance of lipid binding ability. (B) Seven or eight repeats in the ligand binding domains of LDLR (left) and VLDLR (right) were deleted (ΔLBD). (C) An asparagine residue in the repeat 5 and in the repeats 2 to 7 of LDLR was substituted with tyrosine (mut5 and mut2-7). The wild-type and mutants of SR-B1, LDLR and VLDLR were expressed in SR/LD-DKO Huh7 cells by lentiviral vectors. Expressions of these receptors were detected by immunoblotting (middle panels in A and B, right upper panel in C). These cells were infected with HCVcc at an MOI of 1, and intracellular HCV RNA levels at 24 h post-infection were determined by qRT-PCR (lower panels in A and B, right lower panel in C). (D) HCVpp were inoculated into parental, SR/LD-DKO expressing either SR-B1, LDLR or VLDLR and CD81-KO Huh7 cells, and luciferase activities were determined at 48 h post-infection by using a luciferase assay system. In all cases, asterisks indicate significant differences (*P<0.05; **P<0.01) versus the results for cells expressing wild-type receptors.

Previous studies revealed that mutations of S112F and T175A in SR-B1 were observed in patients with high-HDL cholesterol levels in sera [21]. These mutations are located in the large extracellular loop region of SR-B1 and abrogate binding to HDL and uptake of lipid [22]. SR/LD-DKO Huh7 cells expressing either the wild-type or mutant SR-B1 by lentiviral vectors were established. To confirm the cell surface expression of mutants of SR-B1, biotinylated cell surface proteins were purified and examined by immunoblotting (S5A Fig). Although GFP-HA and actin in the cytoplasm were not biotinylated, the wild-type and mutants of lipoprotein receptors were biotinylated and detected in the plasma membrane fractions similar to EGFR used as a plasma membrane marker, suggesting that both wild-type and mutant lipoprotein receptors are similarly expressed on the cell surface. To determine lipoprotein uptake activity, lipid transfer assay was performed in SR/LD-DKO Huh7 cells expressing several mutants by using fluorescent-labeled HDL and LDL (S5D–S5F Fig). Expression of wild-type but not of S112F and T175A mutants facilitates to uptake of HDL and LDL in SR/LD-DKO Huh7 cells. To examine the roles of the lipid uptake machinery in HCV entry, SR/LD-DKO Huh7 cells expressing either the wild-type or mutant SR-B1 were inoculated with HCVcc at an MOI of 1, and intracellular HCV RNA levels were determined by qRT-PCR at 24 h post-infection (Fig 6A). Expression of wild-type SR-B1 but not of the S112F and T175A mutants completely rescued HCV entry, suggesting that lipoprotein binding and lipid uptake of SR-B1 participate in HCV entry.

LDLR and VLDLR have several repeats in the ligand binding domain that is responsible for the uptake of LDL and VLDL. To examine whether these repeats participate in HCV entry, LDLR and VLDLR mutants with these repeats deleted were expressed in SR/LD-DKO Huh7 cells and the intracellular HCV RNA levels were determined upon infection with HCVcc at an MOI of 1 by qRT-PCR at 24 h post-infection (Fig 6B). Lipid transfer assay revealed that these deletions almost abrogated the lipid uptake ability (S5 Fig). Expression of deletion mutants in the repeats domain of LDLR and VLDLR in SR/LD-DKO Huh7 cells failed to recover HCV entry, in contrast to the rescue of entry by the expression of the intact LDLR or VLDLR, suggesting that the repeats in the ligand binding domain of LDLR and VLDLR are important for HCV entry. To rule out the possibility that deletions in the repeat-containing domain caused a loss of original domain structures, point mutants of LDLR were constructed (S5 Fig). A previous study revealed that substitution of asparagine to tyrosine in the repeat 5 and in the repeats from 2 to 7 was crucial for binding to LDL and VLDL [23]. To examine the roles of the binding ability of LDLR to LDL and VLDL in HCV entry, SR/LD-DKO Huh7 cells expressing either wild-type or mutant LDLR by lentiviral vectors were inoculated with HCVcc at an MOI of 1, and intracellular HCV RNA levels were determined by qRT-PCR at 24 h post-infection (Fig 6C). Expression of the wild-type LDLR but not of the mutants rescued HCV entry, suggesting that that ability of LDLR to bind LDL and VLDL is important for HCV entry.

To further examine the involvement of the interaction between viral envelope proteins and the receptors in the lipoprotein receptor-mediated HCV entry, the roles of SR-B1, LDLR and VLDLR in entry of the HCV pseudotype particles (HCVpp) were determined (Fig 6D). HCVpp bearing HCV envelope glycoproteins was generated in 293T cells that were deficient in lipoprotein production. Although CD81 KO Huh7 cells did not show any susceptibility to HCVpp infection, the infectivity of HCVpp to SR/LD-DKO Huh7 cells was comparable to that in SR/LD-DKO Huh7 cells expressing lipoprotein receptor, suggesting that viral particle-associated lipoproteins participate in the lipoprotein receptor-mediated HCV entry.

### Density-dependent entry of LVPs via lipoprotein receptors

**Fig 7:**
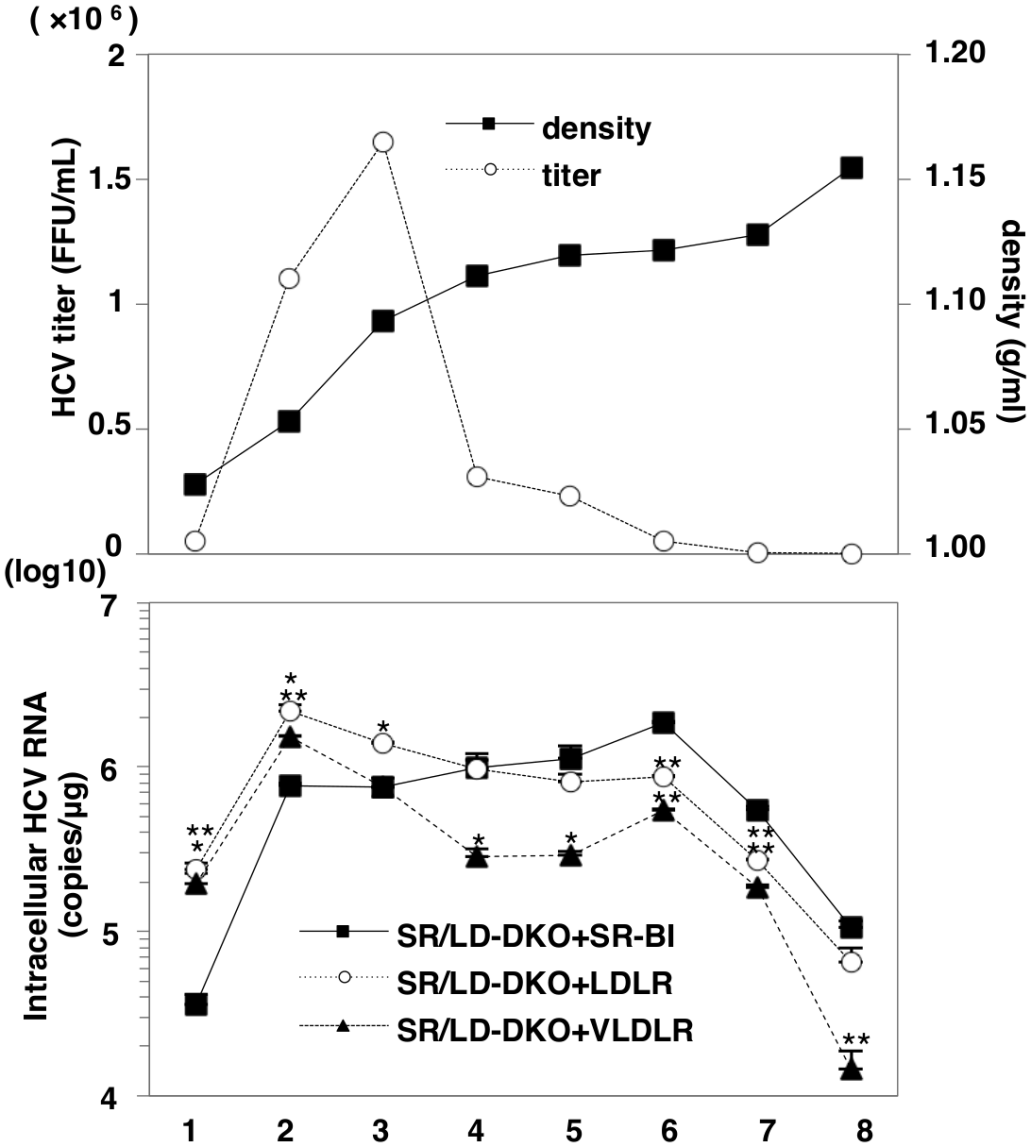
Density-dependent entry of LVPs via lipoprotein receptors. HCV particles in the culture supernatants of Huh7.5.1 cells were harvested at 72 h post-infection with HCVcc and fractionated by using density gradient centrifugation. Infectious titers of each fraction were determined by focus-forming assay and the buoyant density was plotted for each fraction (upper panels). SR/LD-DKO Huh7 cells expressing either SR-B1, LDLR or VLDLR were infected with each fraction and intracellular HCV RNA levels at 24 h post-infection were determined by qRT-PCR (lower panel). Asterisks indicate significant differences (*P<0.05; **P<0.01) versus the results for SR/LD-DKO Huh7 cells expressing SR-B1.

Although buoyant density gradient analyses have shown that viral RNA and infectious particles were broadly distributed at various densities, it is not clear whether the utilization of lipoprotein receptors is associated with viral particle densities. To determine the involvement of LVP density in lipoprotein receptor-mediated HCV entry, HCVcc was fractionated by buoyant density gradient ultracentrifugation and each fraction was inoculated into SR/LD-DKO Huh7 cells expressing SR-B1, LDLR or VLDLR. The infectious titer at 72 h post-infection and intracellular HCV RNA levels at 24 h post-infection in each fraction were determined by focus forming assay and qRT-PCR, respectively (Fig 7). HCV in the high-density and low-density fractions exhibited higher affinity for SR/LD-DKO Huh7 cells complemented with SR-B1 and to those complemented with LDLR and VLDLR, respectively. These results suggest that lipoprotein receptors such as SR-B1, LDLR and VLDLR participate in HCV entry in a manner that is dependent on the density of LVP.

## Discussion

In this study, we demonstrated that HCV-associated lipoproteins are involved in HCV entry via lipoprotein receptors such as SR-B1, LDLR and VLDLR. In addition, our data indicated that these lipoprotein receptors redundantly participate in HCV entry in a manner that is dependent on the density of virion-associated lipoproteins.

Previous studies have shown that inhibition of HCV entry by knockdown of both SR-B1 and LDLR was comparable to that by knockdown of either receptor alone, suggesting that SR-B1 and LDLR independently participate in HCV entry [24]. In this study, we employed gene-knockout techniques with a CRISPR/Cas9 system to obtain data more reliable than that by knockdown experiments and demonstrated that SR-B1 and LDLR have a redundant role in HCV entry (Fig 3).

Several reports have revealed the involvement of lipoproteins in HCV entry via SR-B1 and LDLR. Binding of SR-B1 to HCV particles derived from patient sera was inhibited by the treatment of ApoE and VLDL [25], and lipid transfer activity of SR-B1 was shown to be involved in HCV entry [26, 27]. In addition, HCV entry was inhibited by the treatment with antibodies against LDLR, ApoE and ApoB [28], and the interaction between HCV-associated apolipoproteins and LDLR facilitated efficient HCV entry [12, 29]. On the other hand, Catanese et al. showed that HCVcc efficiently infects Huh7.5 cells in the absence of serum lipoproteins [30]. However, we demonstrated herein that the ligand binding activity of SR-B1, LDLR and VLDLR is crucial for HCV entry, and overexpression of these lipoprotein receptors in SR/LD-DKO Huh7 cells has no effect on infection with HCV pseudotype particles (Fig 6D). In addition, a recent report showed that HCVpp exhibited NPC1L1-independent cell entry and the cholesterol-abundant HCVcc exhibited enhanced NPC1L1-dependent entry, suggesting that virion-associated cholesterol is involved in viral entry via NPC1L1 [10]. Furthermore, knockdown of CIDEB affected entry of HCVcc but not of HCVpp [11]. Both CIDEB and NPC1L1 are involved in the regulation of lipid metabolism, and therefore HCV may utilize lipid metabolism for its cellular entry. The present results suggest that HCV-associated lipoproteins are involved in lipoprotein receptor-mediated entry of HCV.

Although our current data demonstrated that deficiency or expression of SR-B1, LDLR and VLDLR had no effect on entry of HCVpp (Fig 6D), previous study showed that HCVpp entry is dependent on SR-B1[27]. They examined HCVpp assay in rat hepatocarcinoma cells (BRL cells) expressing CD81, CLDN1 and SR-B1 and showed that lipid transfer activity of SR-B1 were required for HCV entry. There is a possibility that other factors can compensate the lipid transfer activity of SR-B1 in Huh7 cells, in contrast to BRL cells. Actually, deficiency of SR-B1 and LDLR cannot block HCV entry completely, compared to CD81 or CLDN1 KO Huh7 cells (Figs 1B and 3A).

In addition to SR-B1 and LDLR, we demonstrated that VLDLR, which is structurally similar to LDLR, plays roles in HCV entry similar to those of SR-B1 and LDLR (Fig 4). Very recently, Ujino et al. showed that HCV utilizes VLDLR for entry independently form CD81, CLDN1 and OCLN-mediated pathway[20], in contrast to our current results suggesting that expression of VLDLR had no effect on entry of HCV in CD81KO Huh7 cells. These discrepancies might be attributable to the difference in experimental procedures. First, Ujino et al. established CD81 KO Huh7.5 cells whereas we utilized Huh7 cells. Second, they applied knockdown to evaluate the role of CLDN1 and OCLN in contrast to knockout in our study. Third, they used untagged VLDLR while we used HA-tagged VLDLR. Last, they used VLDLR variant 2 lacking O-linked sugar domain while we used variant 1. Further studies are needed to clarify the precise roles of VLDLR in HCV entry.

Although we demonstrated that expression of VLDLR in SR/LD-DKO Huh7 cells significantly rescued HCV entry, enhancement in parental and SR-KO Huh7 cells were not significant. There are possibilities that the affinity of HCV to VLDLR is weaker than that to SR-B1 and LDLR. VLDLR is widely expressed in peripheral tissues but not in hepatocytes, in contrast to the abundant expression of SR-B1 and LDLR in the liver. Both SR-B1 and CLDN1 are specifically expressed in the liver, and their expression might be involved in determining the tissue tropism of HCV infection [31]. However, previous reports have shown that CLDN6 and CLDN9 expressed in various tissues play roles in HCV entry into non-hepatic human cells that are comparable to those played by CLDN1 in HCV entry into human hepatic cells [32–34]. Therefore, it might be feasible to speculate that expression of VLDLR and CLDN6/9 enables HCV to be internalized into various non-hepatic tissues, leading to development of the extrahepatic manifestations that sometimes occur in chronic hepatitis C patients.

A number of host factors have been shown to participate in HCV entry into human hepatocytes. Heparan sulfate, LDLR and SR-B1 are thought to mediate the initial attachment of the lipoprotein-associated HCV particles to the cell surface of hepatocytes. After the initial binding, CD81, CLDN1 and OCLN initiate HCV internalization and induce clathrin-mediated endocytosis [32, 35, 36]. A previous study reported that SR-B1 is involved in the early step of HCV entry [37], as seen also in our current study. In addition, we have shown that not only SR-B1 but also LDLR and VLDLR are involved in the binding step of HCV (Fig 5). On the other hand, several reports have shown that SR-B1 is involved in the post-binding step of HCV entry [26, 38]. Although we demonstrated that LDLR and VLDLR can compensate the role of SR-B1 in HCV entry, the roles of LDLR and VLDLR in post-binding step were not evaluated in this study. Previous reports have shown that lipid transfer activities of SR-B1 are required as a post-binding step in HCV entry [26, 27]. Therefore it might be feasible to speculate that other factors such as LDLR and VLDLR are involved in the post-binding step by using lipid transfer activity or that other mechanisms participate in HCV entry in the absence of SR-B1. Further studies are needed to clarify the roles of lipoprotein receptors in the post-binding step.

Wünschmann et al. demonstrated that low-density HCV particles but not intermediate-density particles bound to LDLR-expressing cells [39]. In addition, Thi et al. showed that SR-B1 mediates primary attachment of HCV particles of intermediate density to cells [27]. These data are consistent with our present finding that the lipoprotein receptor usage of HCV is dependent on viral particle density (Fig 7). The apolipoproteins on lipoproteins differ according to the lipoprotein density, and HCV particles derived from patient sera are associated with the exchangeable apolipoproteins ApoA-1, ApoB48, ApoB100, ApoC-1, ApoC-3 and ApoE [40–44], suggesting that LVPs engage high- and low-density lipoprotein receptors during uptake into hepatocytes.

Previous reports have demonstrated the presence of cell-to-cell infection of HCV, which means that HCV particles are directly transmitted to neighboring cells without viral particles production in the extracellular space [45–47]. Although CD81 is a critical factor in cell-free HCV infection, it was shown that CD81 is dispensable for the cell-to-cell spread of HCVcc [48]. Several reports have shown that SR-B1 is involved in not only cell-free but also cell-to-cell infection by using SR-B1 antibodies [38, 49]. In addition, recent reports showed that cell-to-cell infection was affected by siRNA-mediated knockdown and expression of ApoE [50, 51], suggesting that lipoprotein receptors including SR-B1, LDLR and VLDLR redundantly participate in not only cell-free but also cell-to-cell infection of HCV.

Several reports have shown the involvement of lipoprotein receptors in the entry of other viruses. Bovine viral diarrhea virus (BVDV) and GB virus C (GBV-C), members of the *Flaviviridae* family, were reported to use LDLR in entry [28], and the cell surface expression of LDLR was increased upon infection with dengue virus (DENV) [52]. Furthermore, siRNA-mediated knockdown of SR-B1 abolished enhancement of DENV infection through the interaction between SR-B1 and virion-associated ApoA-1 in various cell lines including Huh7 [53], and the core protein of DENV was shown to bind to VLDL via ApoE *in vitro* [54]. Finally, levels of total plasma cholesterol, HDL and LDL were shown to be significantly lower in patients with severe dengue hemorrhagic fever than in mild cases or healthy controls [55, 56], suggesting that lipid metabolism participates in Flavivirus infection, as seen in HCV infection.

Although direct-acting antivirals (DAAs) have been applied in a clinical setting, their use is still limited to severe hepatitis, transplantation, HIV/HCV-co-infection or immune-compromised patients [57]. Viral entry is one of the most important steps in the HCV lifecycle, especially in the reinfection of HCV in the graft after liver transplantation. Treatment with inhibitors of HCV entry might be an attractive strategy to prevent reinfection in the transplanted liver. Previous reports have shown that antibodies against SR-B1 inhibit HCV entry not only *in vitro* but also *in vivo*. ITX5061, a SR-B1 antagonist that inhibits HDL catabolism in the liver by targeting SR-B1, was shown to successfully inhibit HCV entry and spread *in vitro* [58]. Although ITX5061 has entered clinical development and was found to be safe and tolerated in a Phase 1b trial, the viral loads were not significantly reduced [59]. In addition, prophylactic administration of monoclonal antibodies against SR-B1 into uPA-SCID mice prior to xenotransplantation with human liver cells can prevent infection and spread of HCV [60–62]. In spite of the potent efficacy of SR-B1 inhibitors in clinical settings, we showed that deficiencies of SR-B1 achieved only marginal reduction in HCV entry into Huh7 cells. This discrepancy might be explained in either of two ways. First, inhibitors of SR-B1 may affect the functions of other lipoprotein receptors. Second, SR-B1 may play a major role in HCV entry *in vivo*, in contrast to the redundant participation of lipoprotein receptors *in vitro*. In any case, SR-B1 is a promising target for development of novel anti-HCV therapeutics.

In summary, we have shown that the lipoprotein receptors SR-B1, LDLR and VLDLR possess redundant roles in HCV entry through their interaction with the viral-associated lipoprotein.

## Materials and Methods

### Cell lines

All cell lines were cultured at 37°C under the conditions of a humidified atmosphere and 5% CO_2_. The human hepatocellular carcinoma-derived Huh7 and human embryonic kidney-derived 293T cells were maintained in DMEM (Sigma) supplemented with 100U/ml penicillin, 100 μg/ml streptomycin, and 10% fetal calf serum (FCS). The Huh7-derived cell line Huh7.5.1 was kindly provided by F. Chisari. The primary human hepatocyte (PHH) was purchased from PhoenixBio.

### Plasmids

The cDNA clones of SR-B1, LDLR, VLDLR, and AcGFP were inserted between the XhoI and XbaI sites of lentiviral vector pCSII-EF-RfA, which was kindly provided by M. Hijikata, and the resulting plasmids were designated pCSII-EF-SR-B1, pCSII-EF-LDLR, pCSII-EF-LDLR-HA, pCSII-EF-VLDLR-HA, pCSII-EF-AcGFP, and pCSII-EF-GFP-HA respectively. The point mutants of SR-B1 and LDLR and the deletion mutants of LDLR and VLDLR were amplified by PCR and introduced into pCSII-EF. The plasmid GFP-NLS-IPS encodes the green fluorescent protein with a simian virus 40 (SV40) nuclear localization signal fused to IPS-1 residues 462 to 540, which have the site of HCV NS3-4A cleavage and mitochondrial localization sequence [19]. The plasmid pHH-JFH1 encodes a full-length cDNA of the JFH1 strain. pHH-JFH1-E2p7NS2mt contains three adaptive mutations in pHH-JFH1 [63]. pJFH1 encodes full-length cDNA of the JFH1 strain, and pSGR-JFH1 encodes subgenomic cDNA of the JFH1 strain. The secreted nano-luc (Nlucsec) fragment from the pNL1.3 vector (Promega) was replaced with the neomycin gene of pSGR-JFH1 and the resulting plasmid was designated pSGR-JFH1-Nlucsec. The plasmid pX330, which encodes hCas9 and sgRNA, was obtained from Addgene (Addgene plasmid 42230). The fragments of guided RNA targeting the CD81, SR-B1, CLDN1, OCLN and LDLR gene were inserted into the Bbs1 site of pX330 and designated pX330-CD81, pX330-SR-B1, pX330-CLDN1, pX330-OCLN, and pX330-LDLR, respectively. The plasmids used in this study were confirmed by sequencing with an ABI 3130 genetic analyzer (Life Technologies).

### Antibodies

Mouse monoclonal antibody to β-actin was purchased from Sigma. Mouse anti-CD81 antibody was purchased from Santa Cruz Biotechnology. Rabbit anti-SR-B1, OCLN, and CLDN1 antibodies were purchased from NOVUS Biologicals, Proteintech and Life Technologies, respectively. Chicken anti-LDLR antibody was purchased from Abcam. Rat anti-HA antibody was purchased from Roche Diagnostics. Rabbit anti-NS5A antibody was prepared as described previously [64]. Alexa Flour (AF) 488-conjugated anti-rabbit IgG antibody and BODIPY558/568 lipid probe were purchased from Life Technologies. 4’, 6-diamidono-2-phenylindole (DAPI) was purchased from Vector Laboratories, Inc.

### Gene silencing

A small interfering RNA (siRNA) pool targeting LDLR, CD81 and control nontargeting siRNA were purchased from Dharmacon, and transfected into cells using Lipofectamine RNAi MAX (Life Technologies) according to the manufacturer’s protocol.

### Preparation of viruses

pHH-JFH1-E2p7NS2mt, pHH-JFH1 were introduced into Huh7.5.1 cells; HCVcc in the supernatant was collected after serial passages; and infectious titers were determined by a focus-forming assay and expressed in focus-forming units (FFU) [64]. RNA transcribed from pJFH2/AS/mtT4 was electroporated into Huh7.5.1 cells; HCVcc in the supernatant was collected after serial passages; and infectious titers were determined by a focus-forming assay and expressed in FFU. All mouse studied were conducted at Hiroshima University (Hiroshima, Japan) in accordance with the guidelines of the local committee for animal experiments. Chimeric mice transplanted with human hepatocytes were generated as described previously[65]. The experimental protocol was approved by the Ethics Review Committee for Animal Experimentation of the Graduate School of Biomedical Sciences (Hiroshima University). The chimeric mice were infected with a 4 x 10^5^ titer of HCVcc. Serum samples were collected at 2 to 8 weeks after infection. HCV pseudotype particles (HCVpp) containing E1 and E2 glycoproteins of JFH1 were produced as previously described [6, 66].

### Lipofection and lentiviral gene transduction

The lentiviral vectors and ViraPower Lentiviral Packaging Mix (Life Technologies) were co-transfected into 293T cells by Trans IT LT-1 (Mirus), and the supernatants were recovered at 48 h post-transfection. The lentivirus titer was determined by using a Lenti XTM qRT-PCR Titration Kit (Clontech), and expression levels and AcGFP were determined at 48 h post-inoculation.

### Immunoblotting

Cells lysed on ice in lysis buffer (20 mM Tris-HCl [pH7.4], 135 mM NaCl, 1% Triton-X 100, 10% glycerol) supplemented with a protease inhibitor mix (Nacalai Tesque) were boiled in loading buffer and subjected to 5–20% gradient SDS-PAGE. The proteins were transferred to polyvinylidene difluoride membranes (Millipore) and reacted with the appropriate antibodies. The immune complexes were visualized with SuperSignal West Femto Substrate (Pierce) and detected by using an LAS-3000 image analyzer system (Fujifilm).

### Generation of gene-knockout Huh7 cell lines

Huh7 cells were transfected with pX330-CD81, pX330-SR-B1, pX330-CLDN1, pX330-OCLN, and pX330-LDLR by Trans IT LT-1 (Mirus), and single cell clones were established by the single cell isolation technique. To screen for gene-knockout Huh7 cell clones, mutations in target loci were determined by using a Surveyor assay (Transgenomic) according to the manufacturer’s protocol. Frameshift of the genes and deficiencies of protein expressions were confirmed by direct sequencing and immunoblotting analysis, respectively.

### Quantitative RT-PCR

For quantification of HCV-RNA, total RNA was extracted from cells by using a PureLink RNA Mini Kit (Invitrogen), and the first-strand cDNA synthesis and qRT-PCR were performed with a TaqMan RNA-to-C_T_ 1-step Kit and ViiA7 system (Life Technologies), respectively, according to the manufacturer’s protocol. The primers for TaqMan PCR targeted to the noncoding region of HCV RNA were synthesized as previously reported [64]. For quantification of gene expression, the synthesis of the first-stranded cDNA was performed by using a PrimeScript TR reagent Kit (Perfect Real Time) (Takara Bio) and quantitive RT-PCR was performed by using Platinum SYBR Green qRT-PCR SuperMix UDG (Life Technologies) according to the manufacturer’s protocol. The primers sequences for amplification of the SR-B1 gene were the following: forward primer 5’-ACCGCACCTTCCAGTTCCAG-3’ and the reverse primer 5’-ATCACCGCCGCACCCAAG-3’. The primers sequences for amplification of the LDLR gene were the following: forward primer 5’-TGCTCTGATGGAAACTGCATCC-3’ and the reverse primer 5’- AGAGTGTCACATTAACGCAGCC-3’. The primers sequences for amplification of the VLDLR gene were the following: forward primer 5’-CTAGTCAACAACCTGAATGATG-3’ and the reverse primer 5’-AAGAATGGCCCATGCGGCAGAA-3’. The primers sequences for amplification of the GAPDH gene were the following: forward primer 5’-ACCACAGTCCATGCCATCAC-3’ and the reverse primer 5’-TCCACCACCCTGTTGCTGTA-3’. Fluorescent signals were analyzed with the ViiA7 system.

### *In vitro* transcription and RNA transfection

The plasmid pSGR-JFH1, pJFH1 and pSGR-JFH1-Nlucsec were linearized with XbaI, and treated with mung bean exonuclease. The linearized DNA was transcribed *in vitro* by using a MEGAscript T7 kit (Life Technologies) according to the manufacturer's protocol. Capped and polyadenylated firefly luciferase (Fluc) RNAs were synthesized by using a mMESSAGE mMACHINE T7 Ultra kit (Life Technologies) according to the manufacturer’s protocol. The *in vitro* transcribed RNA (5 μg) was electroporated into cells at 5×10^6^ cells/0.4 ml under conditions of 190 V and 950 μF using a Gene Pulser (Bio-Rad) and plated on DMEM containing 10% FCS.

### Colony formation

The medium was replaced with fresh DMEM containing 10% FCS and 1 mg/ml G418 at 24 h post-transfection of transcribed RNA. The remaining colonies were fixed with 4% paraformaldehyde (PFA) and stained with crystal violet at 1 month post-electroporation.

### Cell viability

Cell viability was determined by the Cell Titer-Glo Luminescent Cell Viability Assay (Promega) according to the manufacturer’s protocol and expressed in relative light units (RLU) at 24, 48 and 72 h post-seeding.

### NextBio Body Atlas

The NextBio Body Atlas application presents an aggregated analysis of gene expression across various normal tissues, normal cell types, and cancer cell lines. It enables us to investigate the expression of individual genes as well as gene sets. Samples for Body Atlas data are obtained from publicly available studies that are internally curated, annotated, and processed. Body Atlas measurements are generated from all available RNA expression studies that used Affymetrix U133 Plus or U133A Genechip arrays for human studies. The results from 128 human tissue samples were incorporated from 1,067 arrays; 157 human cell types from 1,474 arrays and 359 human cancer cell lines from 376 arrays. Gene queries return a list of relevant tissues or cell types rank-ordered by absolute gene expression and grouped by body systems or across all body systems. In the current analysis, we determined the expression levels of the SR-B1, LDLR and VLDLR in tissues. The details of the analysis protocol developed by NextBio were described previously [67].

### Buoyant density gradient analysis

Culture supernatants of Huh7.5.1 cells infected with HCVcc at 72 h post-infection were passed through 0.45-μm-pore-size filters and concentrated by a Spin-X Concentrator (100,000-molecular-weight cutoff column; Corning, Lowell, MA). One milliliter of concentrated sample was layered onto the top of a linear gradient formed from 10% to 40% of OptiPrep (Axis-Shield PoC) in PBS and spun at 35,000 rpm for 16 h at 4°C by using an SW41-Ti rotor (Beckman Coulter). Each fraction collected from the top was analyzed by qRT-PCR and focus-forming assay.

### Binding assay

HCVcc was bound to the cells for 1 h at 4°C, washed three times with PBS, and HCV RNA levels were determined by qRT-PCR immediately following binding.

### Immunofluorescence assay

For lipid droplet staining, cells were incubated in medium containing 10μg/ml BODIPY for 20 min at 37°C, washed with prewarmed fresh medium, and incubated for 20 min at 37°C. Cells cultured on glass slides were fixed with 4% PFA in PBS at room temperature for 30 min. Cell nuclei were stained with DAPI. Cells were observed with a FluoView FV1000 laser scanning confocal microscope (Olympus). Quantification of images was performed with ImageJ software (National Institutes of Health).

### Purification of cell surface proteins

Cell surface proteins were biotinylated and purified by Cell Surface Protein Biotynylation and Purification Kit (Thermo Fisher Scientific) according to the manufacturer’s protocol. Total cell lysates and biotynylated proteins were applied in immunoblotting analysis.

### Lipid transfer assay

HDL and LDL labeled with 1,1’-Dioctadecyl-3,3,3,3’-tetramethylindocarbocyanine percholorate (DiI-HDL and DiI-LDL) were purchased from Alfa Aesar. Cells were washed with DMEM containing 0.5% fatty acid-free bovine serum albumin (Sigma) (medium A), and medium A containing 5μg DiI-HDL or DiI-LDL was added to each well. After incubation for 2 h at 37°C, cells were washed twice with PBS and observed with a FluoView FV1000 laser scanning confocal microscope. HDL uptake was determined by using HDL Uptake Assay Kit (Bio Vision) according to the manufacturer’s protocol. LDL uptake was determined after incubation with medium containing 10μg/ml BODIPY FL LDL (Thermo Fisher) without FCS for 24 h at 37°C by using PowerScanHT (DS Pharma Biomedical) according to the manufacturer’s protocol.

### Statistical analysis

The data for statistical analyses are the averages of three independent experiments. Results were expressed as the means ±standard deviations. The significance of differences in the means was determined by Student’s *t*-test.

## Supporting Information

S1 Fig Deletion and insertion of the sequences in knockout cells. (A) The characters in red indicate sequences of the CRISPR/Cas9 system targeting CD81, SR-B1, CLDN1, OCDN, or LDLR, and the PAM sequences are boxed. Gene knockout by sequence modification in all alleles of the CD81, SR-B1, CLDN1, OCDN or LDLR gene in knockout cell lines is shown. Dotted lines and characters in brackets indicate deletion and insertion of sequences, respectively. (B) Effects of gene knockouts on cell viability were determined by using Cell Titer-Glo Luminescent Cell Viability Assay. Equal amounts of parental, CD81 KO, SR-KO, CLDN1 KO, OCLN KO, LD-KO and SR/LD-DKO Huh7 cells were seeded and relative light units (RLU) were determined at 24, 48, 72 h post-seeding (upper panel). Lipid droplets and cell nuclei in parental, CD81 KO, SR-KO, CLDN1 KO, OCLN KO, LD-KO and SR/LD-DKO Huh7 cells were stained by BODIPY and DAPI, respectively (middle panel). The mean numbers of lipid droplet per cell were determined by ImageJ quantification (lower panel).
(TIF)

S2 Fig SR-B1 is dispensable for HCV entry into Huh7.5.1 cells. (A) The characters in red indicate sequences of the CRISPR/Cas9 system targeting SR-B1, and the PAM sequences are boxed. Gene knockout by sequence modification in all alleles of the SR-B1 gene in knockout cell lines is shown. Dotted lines and characters in brackets indicate deletion and insertion of sequences, respectively. (B) Expressions of SR-B1 in parental and SR-B1 KO Huh7.5.1 cells were determined by immunoblotting analysis (upper panel). Cells were infected with HCVcc at an MOI of 1, and intracellular HCV RNA levels at 24 h post-infection were determined by qRT-PCR (lower panel). Asterisks indicate significant differences (*P<0.05; **P<0.01) versus the results for Huh7.5.1 cells.
(TIF)

S3 Fig SR-B1 and LDLR are not involved in replication of HCV. (A) A subgenomic HCV RNA replicon of the JFH1 strain was electroporated into SR-KO and LD-KO Huh7 cells with/without expression of SR-B1 or LDLR by lentiviral vector, and the colonies were stained with crystal violet at 1 month post-electroporation after selection with 1 mg/mL of G418. (B) *In vitro-*transcribed JFH1-Nlucsec RNA was electroporated into parental, SR-KO, LD-KO and SR/LD-DKO Huh7 cells together with firefly luciferase (Fluc) RNA as an internal control, and NlucSec activity in the culture supernatants at 24, 48, 72 h post-electroporation was determined after standardization with Fluc activity. (C) Each three clones derived from parental, SR-KO, LD-KO and SR/LD-DKO Huh7 SGR cells were subjected to qRT-PCR after extraction of total RNA (upper panel) and to immunoblotting using anti-NS5A antibody (lower panel). (D) A full-length HCV RNA of JFH1 strain was electroporated into SR/LD-DKO Huh7 cells expressing SR-B1 or LDLR and the production of infectious HCV particles at 24 and 36 h post-electropolation was determined by focus forming assay.
(TIF)

S4 Fig VLDLR has a similar role with SR-B1 and LDLR in HCV entry. VLDLR (variant 1 or 2) or CD81 were exogenously expressed in SR/LD-DKO and CD81 KO Huh7 cells by infection with lentiviral vectors. Expressions of receptors in these cells were determined by immunoblotting analysis (upper panel). Cells were infected with HCVcc at an MOI of 1 and intracellular HCV RNA levels were determined at 24, 48, 72 h post-infection by qRT-PCR (middle panel). Cells were infected with HCVcc at an MOI of 1 and subjected to immunofluorescence analyses by using antibodies against NS5A at 72 h post-infection (lower panel).
(TIF)

S5 Fig Cell surface expression of lipoprotein receptors. S112F- and T175A-SR-B1 missing lipid binding ability (A), ΔLBD of LDLR and VLDLR deleted the ligand binding domains (B), and mut5 and mut2-7 in which asparagine residues in the repeat 5 and in the repeats 2 to 7 of LDLR were substituted with tyrosine (C) were generated. The wild-type and these mutants of SR-B1, LDLR and VLDLR were expressed in SR/LD-DKO Huh7 cells by lentiviral vectors and cell surface proteins were biotinylated and purified by Cell Surface Protein Biotynylation and Purification Kit. Expressions of the repoprotein receptors, EGFR and actin in whole cell lysate and purified proteins were examined by immunoblotting. EGFR and actin were used as a marker of membrane and cytosolic protein, respectively. (D) Lipid uptake activities of mutants of lipoprotein receptors were determined by using lipid transfer assay. Parental Huh7 cells and SR/LD-DKO Huh7 cells expressing either wild-type or mutants of SR-B1, LDLR and VLDLR were incubated with DMEM containing 0.5% fatty acid-free bovine serum albumin and either 5μg DiI-HDL or DiI-LDL for 2h at 37°C, washed twice with PBS, and examined by a laser scanning confocal microscope. HDL (E) and LDL (F) uptake activities of parental Huh7 cells and SR/LD-DKO Huh7 cells expressing either wild-type or mutants of SR-B1 were determined by using HDL Uptake Assay Kit and PowerScanHT, respectively. In all cases, asterisks indicate significant differences (*P<0.05; **P<0.01) versus the results for Huh7 cells.
(TIF)

## References

[1] Maasoumy B, Wedemeyer H. Natural history of acute and chronic hepatitis C. Best Pract Res Clin Gastroenterol. 2012;26(4):401–412. 10.1016/j.bpg.2012.09.009 . 23199500

[2] Jacobson IM, McHutchison JG, Dusheiko G, Di Bisceglie AM, Reddy KR, Bzowej NH, et al Telaprevir for previously untreated chronic hepatitis C virus infection. N Engl J Med. 2011;364(25):2405–2516. 10.1056/NEJMoa1012912 . 21696307

[3] Pawlotsky JM, Flisiak R, Sarin SK, Rasenack J, Piratvisuth T, Chuang WL, et al Alisporivir plus ribavirin, interferon-free or in combination with peg-interferon, for HCV genotype 2 or 3 infection. Hepatology. 2015 10.1002/hep.27960 . 26118427

[4] Pileri P, Uematsu Y, Campagnoli S, Galli G, Falugi F, Petracca R, et al Binding of hepatitis C virus to CD81. Science. 1998;282(5390):938–941. . 9794763 10.1126/science.282.5390.938

[5] Scarselli E, Ansuini H, Cerino R, Roccasecca RM, Acali S, Filocamo G, et al The human scavenger receptor class B type I is a novel candidate receptor for the hepatitis C virus. EMBO J. 2002;21(19):5017–5025. 12356718 10.1093/emboj/cdf529 PMC129051

[6] Bartosch B, Dubuisson J, Cosset FL. Infectious hepatitis C virus pseudo-particles containing functional E1-E2 envelope protein complexes. J Exp Med. 2003;197(5):633–642. 12615904 10.1084/jem.20021756 PMC2193821

[7] Evans MJ, von Hahn T, Tscherne DM, Syder AJ, Panis M, Wölk B, et al Claudin-1 is a hepatitis C virus co-receptor required for a late step in entry. Nature. 2007;446(7137):801–805. 10.1038/nature05654 . 17325668

[8] Ploss A, Evans MJ, Gaysinskaya VA, Panis M, You H, de Jong YP, et al Human occludin is a hepatitis C virus entry factor required for infection of mouse cells. Nature. 2009;457(7231):882–886. 10.1038/nature07684 19182773 PMC2762424

[9] Lupberger J, Zeisel MB, Xiao F, Thumann C, Fofana I, Zona L, et al EGFR and EphA2 are host factors for hepatitis C virus entry and possible targets for antiviral therapy. Nat Med. 2011;17(5):589–595. 10.1038/nm.2341 21516087 PMC3938446

[10] Sainz B, Barretto N, Martin DN, Hiraga N, Imamura M, Hussain S, et al Identification of the Niemann-Pick C1-like 1 cholesterol absorption receptor as a new hepatitis C virus entry factor. Nat Med. 2012;18(2):281–285. 10.1038/nm.2581 22231557 PMC3530957

[11] Wu X, Lee EM, Hammack C, Robotham JM, Basu M, Lang J, et al Cell death-inducing DFFA-like effector b is required for hepatitis C virus entry into hepatocytes. J Virol. 2014;88(15):8433–8444. 10.1128/JVI.00081-14 24829338 PMC4135929

[12] André P, Komurian-Pradel F, Deforges S, Perret M, Berland JL, Sodoyer M, et al Characterization of low- and very-low-density hepatitis C virus RNA-containing particles. J Virol. 2002;76(14):6919–6928. 12072493 10.1128/JVI.76.14.6919-6928.2002 PMC136313

[13] Nielsen SU, Bassendine MF, Burt AD, Martin C, Pumeechockchai W, Toms GL. Association between hepatitis C virus and very-low-density lipoprotein (VLDL)/LDL analyzed in iodixanol density gradients. J Virol. 2006;80(5):2418–2428. 10.1128/JVI.80.5.2418-2428.2006 16474148 PMC1395398

[14] Porteus MH, Carroll D. Gene targeting using zinc finger nucleases. Nat Biotechnol. 2005;23(8):967–973. 10.1038/nbt1125 . 16082368

[15] Zhang F, Cong L, Lodato S, Kosuri S, Church GM, Arlotta P. Efficient construction of sequence-specific TAL effectors for modulating mammalian transcription. Nat Biotechnol. 2011;29(2):149–153. 10.1038/nbt.1775 21248753 PMC3084533

[16] Mali P, Yang L, Esvelt KM, Aach J, Guell M, DiCarlo JE, et al RNA-guided human genome engineering via Cas9. Science. 2013;339(6121):823–826. 10.1126/science.1232033 23287722 PMC3712628

[17] Wang H, Yang H, Shivalila CS, Dawlaty MM, Cheng AW, Zhang F, et al One-step generation of mice carrying mutations in multiple genes by CRISPR/Cas-mediated genome engineering. Cell. 2013;153(4):910–918. 10.1016/j.cell.2013.04.025 23643243 PMC3969854

[18] Cho SW, Kim S, Kim JM, Kim JS. Targeted genome engineering in human cells with the Cas9 RNA-guided endonuclease. Nat Biotechnol. 2013;31(3):230–232. 10.1038/nbt.2507 . 23360966

[19] Jones CT, Catanese MT, Law LM, Khetani SR, Syder AJ, Ploss A, et al Real-time imaging of hepatitis C virus infection using a fluorescent cell-based reporter system. Nat Biotechnol. 2010;28(2):167–171. 10.1038/nbt.1604 20118917 PMC2828266

[20] Ujino S, Nishitsuji H, Hishiki T, Sugiyama K, Takaku H, Shimotohno K. Hepatitis C virus utilizes VLDLR as a novel entry pathway. Proc Natl Acad Sci U S A. 2016;113(1):188–193. 10.1073/pnas.1506524113 26699506 PMC4711846

[21] Brunham LR, Tietjen I, Bochem AE, Singaraja RR, Franchini PL, Radomski C, et al Novel mutations in scavenger receptor BI associated with high HDL cholesterol in humans. Clin Genet. 2011;79(6):575–581. 10.1111/j.1399-0004.2011.01682.x . 21480869

[22] Chadwick AC, Sahoo D. Functional characterization of newly-discovered mutations in human SR-BI. PLoS One. 2012;7(9):e45660 10.1371/journal.pone.0045660 23029167 PMC3448639

[23] Russell DW, Brown MS, Goldstein JL. Different combinations of cysteine-rich repeats mediate binding of low density lipoprotein receptor to two different proteins. J Biol Chem. 1989;264(36):21682–21688. . 2600087

[24] Hishiki T, Shimizu Y, Tobita R, Sugiyama K, Ogawa K, Funami K, et al Infectivity of hepatitis C virus is influenced by association with apolipoprotein E isoforms. J Virol. 2010;84(22):12048–12057. 10.1128/JVI.01063-10 20826689 PMC2977863

[25] Maillard P, Huby T, Andréo U, Moreau M, Chapman J, Budkowska A. The interaction of natural hepatitis C virus with human scavenger receptor SR-BI/Cla1 is mediated by ApoB-containing lipoproteins. FASEB J. 2006;20(6):735–737. 10.1096/fj.05-4728fje . 16476701

[26] Zeisel MB, Koutsoudakis G, Schnober EK, Haberstroh A, Blum HE, Cosset FL, et al Scavenger receptor class B type I is a key host factor for hepatitis C virus infection required for an entry step closely linked to CD81. Hepatology. 2007;46(6):1722–1731. 10.1002/hep.21994 . 18000990

[27] Dao Thi VL, Granier C, Zeisel MB, Guérin M, Mancip J, Granio O, et al Characterization of hepatitis C virus particle subpopulations reveals multiple usage of the scavenger receptor BI for entry steps. J Biol Chem. 2012;287(37):31242–31257. 10.1074/jbc.M112.365924 22767607 PMC3438956

[28] Agnello V, Abel G, Elfahal M, Knight GB, Zhang QX. Hepatitis C virus and other flaviviridae viruses enter cells via low density lipoprotein receptor. Proc Natl Acad Sci U S A. 1999;96(22):12766–12771. 10535997 10.1073/pnas.96.22.12766 PMC23090

[29] Owen DM, Huang H, Ye J, Gale M. Apolipoprotein E on hepatitis C virion facilitates infection through interaction with low-density lipoprotein receptor. Virology. 2009;394(1):99–108. 10.1016/j.virol.2009.08.037 19751943 PMC2767442

[30] Catanese MT, Graziani R, von Hahn T, Moreau M, Huby T, Paonessa G, et al High-avidity monoclonal antibodies against the human scavenger class B type I receptor efficiently block hepatitis C virus infection in the presence of high-density lipoprotein. J Virol. 2007;81(15):8063–8071. 10.1128/JVI.00193-07 17507483 PMC1951280

[31] Ploss A, Evans MJ. Hepatitis C virus host cell entry. Curr Opin Virol. 2012;2(1):14–19. 10.1016/j.coviro.2011.12.007 22440961 PMC3311996

[32] Zheng A, Yuan F, Li Y, Zhu F, Hou P, Li J, et al Claudin-6 and claudin-9 function as additional coreceptors for hepatitis C virus. J Virol. 2007;81(22):12465–12471. 10.1128/JVI.01457-07 17804490 PMC2169001

[33] Meertens L, Bertaux C, Cukierman L, Cormier E, Lavillette D, Cosset FL, et al The tight junction proteins claudin-1, -6, and -9 are entry cofactors for hepatitis C virus. J Virol. 2008;82(7):3555–3560. 10.1128/JVI.01977-07 18234789 PMC2268462

[34] Haid S, Grethe C, Dill MT, Heim M, Kaderali L, Pietschmann T. Isolate-dependent use of claudins for cell entry by hepatitis C virus. Hepatology. 2014;59(1):24–34. 10.1002/hep.26567 . 23775920

[35] Bertaux C, Dragic T. Different domains of CD81 mediate distinct stages of hepatitis C virus pseudoparticle entry. J Virol. 2006;80(10):4940–4948. 10.1128/JVI.80.10.4940-4948.2006 16641285 PMC1472091

[36] Sourisseau M, Michta ML, Zony C, Israelow B, Hopcraft SE, Narbus CM, et al Temporal analysis of hepatitis C virus cell entry with occludin directed blocking antibodies. PLoS Pathog. 2013;9(3):e1003244 10.1371/journal.ppat.1003244 23555257 PMC3605286

[37] Catanese MT, Ansuini H, Graziani R, Huby T, Moreau M, Ball JK, et al Role of scavenger receptor class B type I in hepatitis C virus entry: kinetics and molecular determinants. J Virol. 2010;84(1):34–43. 10.1128/JVI.02199-08 19828610 PMC2798406

[38] Zahid MN, Turek M, Xiao F, Thi VL, Guérin M, Fofana I, et al The postbinding activity of scavenger receptor class B type I mediates initiation of hepatitis C virus infection and viral dissemination. Hepatology. 2013;57(2):492–504. 10.1002/hep.26097 . 23081796

[39] Wünschmann S, Medh JD, Klinzmann D, Schmidt WN, Stapleton JT. Characterization of hepatitis C virus (HCV) and HCV E2 interactions with CD81 and the low-density lipoprotein receptor. J Virol. 2000;74(21):10055–10062. 11024134 10.1128/jvi.74.21.10055-10062.2000 PMC102044

[40] Catanese MT, Uryu K, Kopp M, Edwards TJ, Andrus L, Rice WJ, et al Ultrastructural analysis of hepatitis C virus particles. Proc Natl Acad Sci U S A. 2013;110(23):9505–9510. 10.1073/pnas.1307527110 23690609 PMC3677472

[41] Chang KS, Jiang J, Cai Z, Luo G. Human apolipoprotein e is required for infectivity and production of hepatitis C virus in cell culture. J Virol. 2007;81(24):13783–13793. 10.1128/JVI.01091-07 17913825 PMC2168882

[42] Gastaminza P, Cheng G, Wieland S, Zhong J, Liao W, Chisari FV. Cellular determinants of hepatitis C virus assembly, maturation, degradation, and secretion. J Virol. 2008;82(5):2120–2129. 10.1128/JVI.02053-07 18077707 PMC2258938

[43] Meunier JC, Russell RS, Engle RE, Faulk KN, Purcell RH, Emerson SU. Apolipoprotein c1 association with hepatitis C virus. J Virol. 2008;82(19):9647–9656. 10.1128/JVI.00914-08 18667498 PMC2546963

[44] Sun HY, Lin CC, Lee JC, Wang SW, Cheng PN, Wu IC, et al Very low-density lipoprotein/lipo-viro particles reverse lipoprotein lipase-mediated inhibition of hepatitis C virus infection via apolipoprotein C-III. Gut. 2013;62(8):1193–1203. 10.1136/gutjnl-2011-301798 . 22689516

[45] Chang M, Williams O, Mittler J, Quintanilla A, Carithers RL, Perkins J, et al Dynamics of hepatitis C virus replication in human liver. Am J Pathol. 2003;163(2):433–444. 10.1016/S0002-9440(10)63673-5 12875965 PMC1868229

[46] Timpe JM, Stamataki Z, Jennings A, Hu K, Farquhar MJ, Harris HJ, et al Hepatitis C virus cell-cell transmission in hepatoma cells in the presence of neutralizing antibodies. Hepatology. 2008;47(1):17–24. 10.1002/hep.21959 . 17941058

[47] Wieland S, Makowska Z, Campana B, Calabrese D, Dill MT, Chung J, et al Simultaneous detection of hepatitis C virus and interferon stimulated gene expression in infected human liver. Hepatology. 2014;59(6):2121–2130. 10.1002/hep.26770 24122862 PMC3975814

[48] Witteveldt J, Evans MJ, Bitzegeio J, Koutsoudakis G, Owsianka AM, Angus AG, et al CD81 is dispensable for hepatitis C virus cell-to-cell transmission in hepatoma cells. J Gen Virol. 2009;90(Pt 1):48–58. 10.1099/vir.0.006700-0 19088272 PMC2885024

[49] Catanese MT, Loureiro J, Jones CT, Dorner M, von Hahn T, Rice CM. Different requirements for scavenger receptor class B type I in hepatitis C virus cell-free versus cell-to-cell transmission. J Virol. 2013;87(15):8282–8293. 10.1128/JVI.01102-13 23698298 PMC3719822

[50] Hueging K, Doepke M, Vieyres G, Bankwitz D, Frentzen A, Doerrbecker J, et al Apolipoprotein E codetermines tissue tropism of hepatitis C virus and is crucial for viral cell-to-cell transmission by contributing to a postenvelopment step of assembly. J Virol. 2014;88(3):1433–1446. 10.1128/JVI.01815-13 24173232 PMC3911621

[51] Gondar V, Molina-Jiménez F, Hishiki T, García-Buey L, Koutsoudakis G, Shimotohno K, et al Apolipoprotein E, but Not Apolipoprotein B, Is Essential for Efficient Cell-to-Cell Transmission of Hepatitis C Virus. J Virol. 2015;89(19):9962–9973. 10.1128/JVI.00577-15 . 26202245 PMC4577890

[52] Soto-Acosta R, Mosso C, Cervantes-Salazar M, Puerta-Guardo H, Medina F, Favari L, et al The increase in cholesterol levels at early stages after dengue virus infection correlates with an augment in LDL particle uptake and HMG-CoA reductase activity. Virology. 2013;442(2):132–147. 10.1016/j.virol.2013.04.003 . 23642566

[53] Li Y, Kakinami C, Li Q, Yang B, Li H. Human apolipoprotein A-I is associated with dengue virus and enhances virus infection through SR-BI. PLoS One. 2013;8(7):e70390 10.1371/journal.pone.0070390 23894648 PMC3722190

[54] Faustino AF, Guerra GM, Huber RG, Hollmann A, Domingues MM, Barbosa GM, et al Understanding dengue virus capsid protein disordered N-Terminus and pep14-23-based inhibition. ACS Chem Biol. 2015;10(2):517–526. 10.1021/cb500640t . 25412346

[55] van Gorp EC, Suharti C, Mairuhu AT, Dolmans WM, van Der Ven J, Demacker PN, et al Changes in the plasma lipid profile as a potential predictor of clinical outcome in dengue hemorrhagic fever. Clin Infect Dis. 2002;34(8):1150–1153. 10.1086/339539 . 11915007

[56] Suvarna JC, Rane PP. Serum lipid profile: a predictor of clinical outcome in dengue infection. Trop Med Int Health. 2009;14(5):576–585. 10.1111/j.1365-3156.2009.02261.x . 19309479

[57] Liang CM, Hu TH, Lu SN, Hung CH, Huang CM, Wang JH, et al Role of hepatitis C virus substitutions and interleukin-28B polymorphism on response to peginterferon plus ribavirin in a prospective study of response-guided therapy. J Viral Hepat. 2013;20(11):761–769. 10.1111/jvh.12097 . 24168255

[58] Syder AJ, Lee H, Zeisel MB, Grove J, Soulier E, Macdonald J, et al Small molecule scavenger receptor BI antagonists are potent HCV entry inhibitors. J Hepatol. 2011;54(1):48–55. 10.1016/j.jhep.2010.06.024 . 20932595

[59] Sulkowski MS, Kang M, Matining R, Wyles D, Johnson VA, Morse GD, et al Safety and antiviral activity of the HCV entry inhibitor ITX5061 in treatment-naive HCV-infected adults: a randomized, double-blind, phase 1b study. J Infect Dis. 2014;209(5):658–667. 10.1093/infdis/jit503 24041792 PMC3923538

[60] Meuleman P, Catanese MT, Verhoye L, Desombere I, Farhoudi A, Jones CT, et al A human monoclonal antibody targeting scavenger receptor class B type I precludes hepatitis C virus infection and viral spread in vitro and in vivo. Hepatology. 2012;55(2):364–372. 10.1002/hep.24692 21953761 PMC3262867

[61] Lacek K, Vercauteren K, Grzyb K, Naddeo M, Verhoye L, Słowikowski MP, et al Novel human SR-BI antibodies prevent infection and dissemination of HCV in vitro and in humanized mice. J Hepatol. 2012;57(1):17–23. 10.1016/j.jhep.2012.02.018 . 22414763

[62] Vercauteren K, Van Den Eede N, Mesalam AA, Belouzard S, Catanese MT, Bankwitz D, et al Successful anti-scavenger receptor class B type I (SR-BI) monoclonal antibody therapy in humanized mice after challenge with HCV variants with in vitro resistance to SR-BI-targeting agents. Hepatology. 2014;60(5):1508–1518. 10.1002/hep.27196 24797654 PMC4211977

[63] Russell RS, Meunier JC, Takikawa S, Faulk K, Engle RE, Bukh J, et al Advantages of a single-cycle production assay to study cell culture-adaptive mutations of hepatitis C virus. Proc Natl Acad Sci U S A. 2008;105(11):4370437–5. 10.1073/pnas.0800422105 18334634 PMC2393785

[64] Fukuhara T, Kambara H, Shiokawa M, Ono C, Katoh H, Morita E, et al Expression of microRNA miR-122 facilitates an efficient replication in nonhepatic cells upon infection with hepatitis C virus. J Virol. 2012;86(15):7918–7933. 10.1128/JVI.00567-12 22593164 PMC3421686

[65] Tateno C, Yoshizane Y, Saito N, Kataoka M, Utoh R, Yamasaki C, et al Near completely humanized liver in mice shows human-type metabolic responses to drugs. Am J Pathol. 2004;165(3):901–912. 10.1016/S0002-9440(10)63352-4 15331414 PMC1618591

[66] Bartosch B, Vitelli A, Granier C, Goujon C, Dubuisson J, Pascale S, et al Cell entry of hepatitis C virus requires a set of co-receptors that include the CD81 tetraspanin and the SR-B1 scavenger receptor. J Biol Chem. 2003;278(43):41624–41630. 10.1074/jbc.M305289200 . 12913001

[67] Kupershmidt I, Su QJ, Grewal A, Sundaresh S, Halperin I, Flynn J, et al Ontology-based meta-analysis of global collections of high-throughput public data. PLoS One. 2010;5(9). 10.1371/journal.pone.0013066 20927376 PMC2947508

