# Supplementary figures and images for: Lipoprotein Receptors Redundantly Participate in Entry of Hepatitis C Virus
Source: PLoS Pathog. 2016 May 6;12(5):e1005610. doi: 10.1371/journal.ppat.1005610 (PMC4859476; doi:10.1371/journal.ppat.1005610)

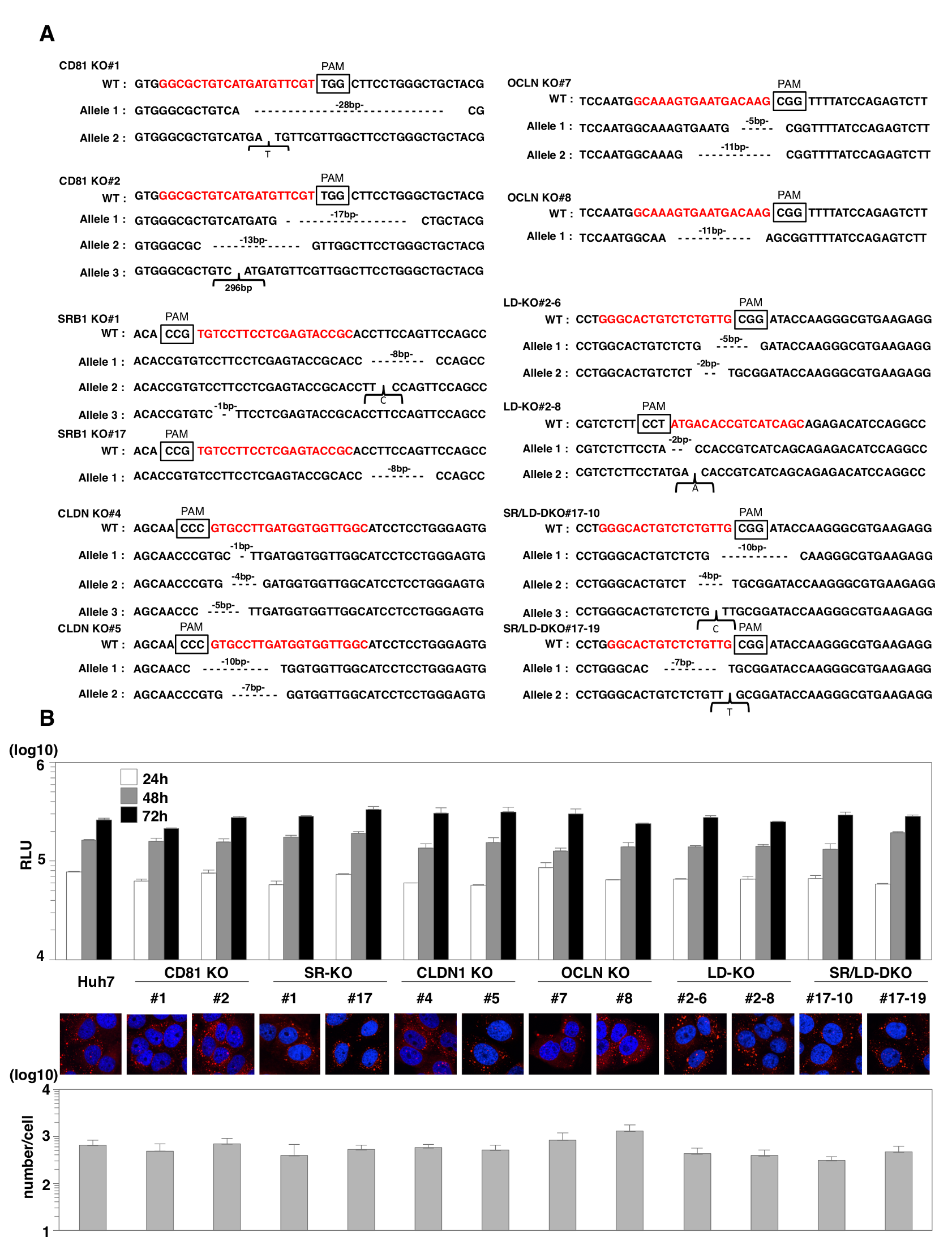

Supplement: S1 Fig — (A) The characters in red indicate sequences of the CRISPR/Cas9 system targeting CD81, SR-B1, CLDN1, OCDN, or LDLR, and the PAM sequences are boxed. Gene knockout by sequence modification in all alleles of the CD81, SR-B1, CLDN1, OCDN or LDLR gene in knockout cell lines is shown. Dotted lines and characters in brackets indicate deletion and insertion of sequences, respectively. (B) Effects of gene knockouts on cell viability were determined by using Cell Titer-Glo Luminescent Cell Viability Assay. Equal amounts of parental, CD81 KO, SR-KO, CLDN1 KO, OCLN KO, LD-KO and SR/LD-DKO Huh7 cells were seeded and relative light units (RLU) were determined at 24, 48, 72 h post-seeding (upper panel). Lipid droplets and cell nuclei in parental, CD81 KO, SR-KO, CLDN1 KO, OCLN KO, LD-KO and SR/LD-DKO Huh7 cells were stained by BODIPY and DAPI, respectively (middle panel). The mean numbers of lipid droplet per cell were determined by ImageJ quantification (lower panel). (TIF) [file ppat.1005610.s001.tif]

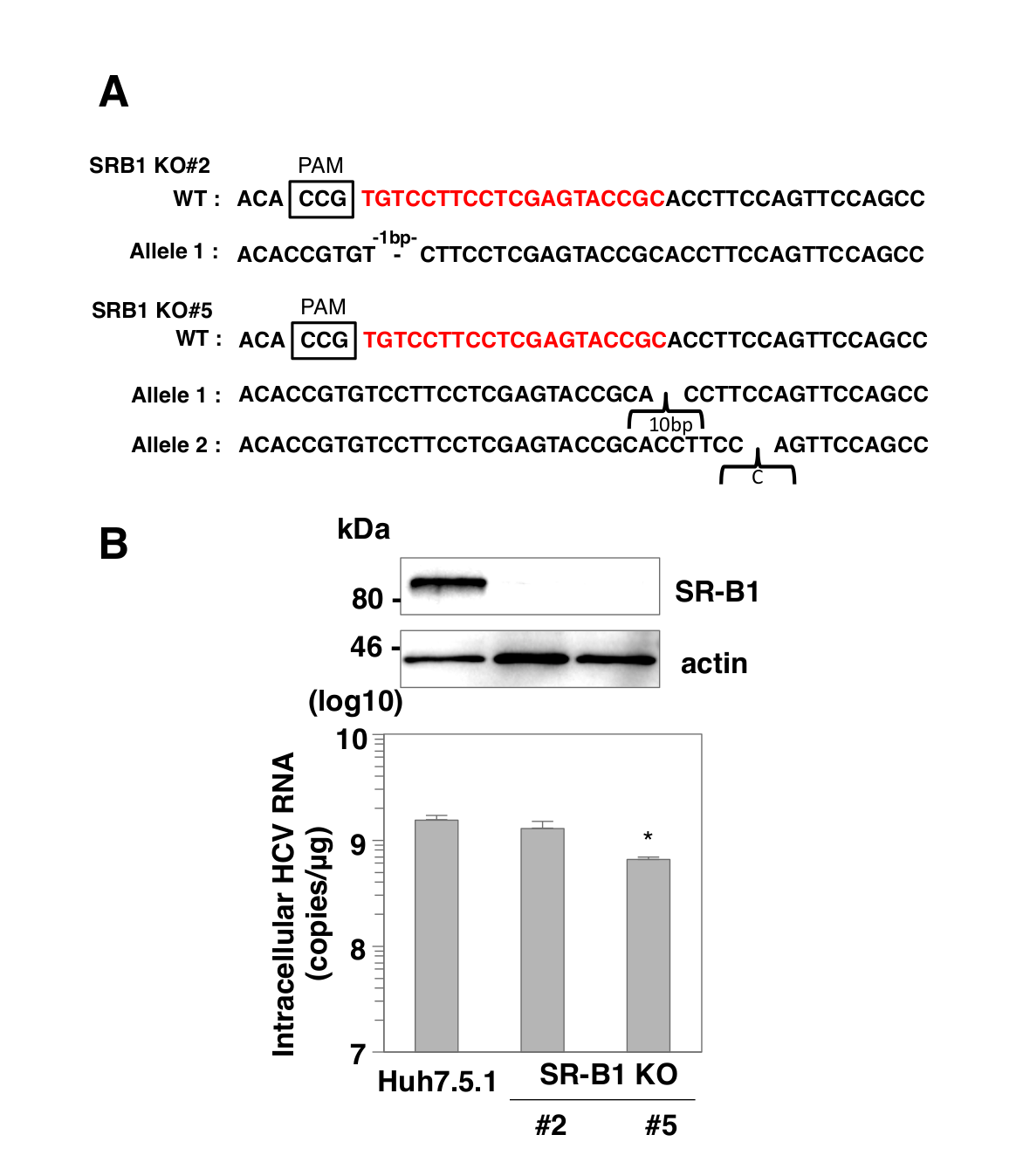

Supplement: S2 Fig — (A) The characters in red indicate sequences of the CRISPR/Cas9 system targeting SR-B1, and the PAM sequences are boxed. Gene knockout by sequence modification in all alleles of the SR-B1 gene in knockout cell lines is shown. Dotted lines and characters in brackets indicate deletion and insertion of sequences, respectively. (B) Expressions of SR-B1 in parental and SR-B1 KO Huh7.5.1 cells were determined by immunoblotting analysis (upper panel). Cells were infected with HCVcc at an MOI of 1, and intracellular HCV RNA levels at 24 h post-infection were determined by qRT-PCR (lower panel). Asterisks indicate significant differences (*P<0.05; **P<0.01) versus the results for Huh7.5.1 cells. (TIF) [file ppat.1005610.s002.tif]

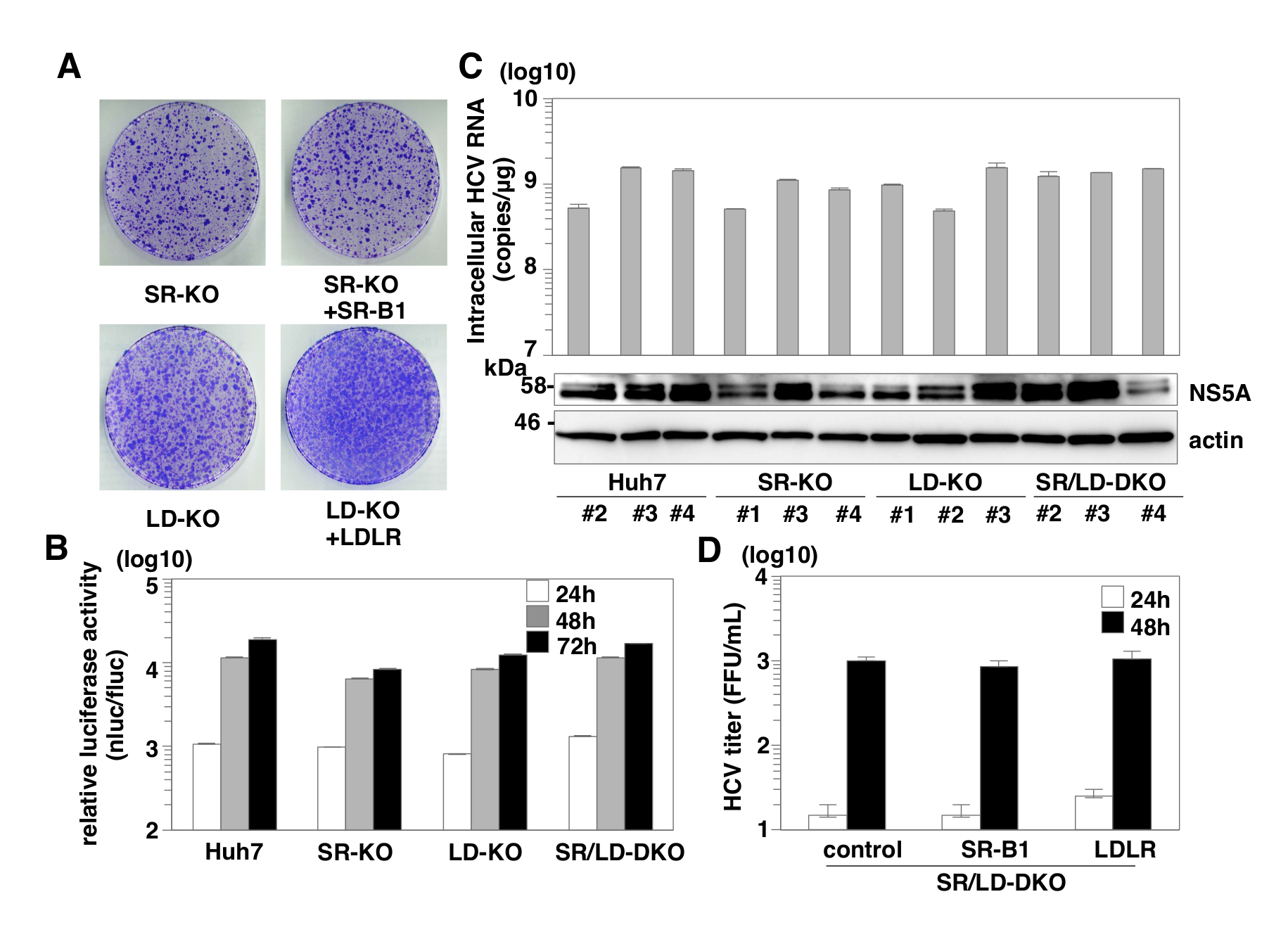

Supplement: S3 Fig — (A) A subgenomic HCV RNA replicon of the JFH1 strain was electroporated into SR-KO and LD-KO Huh7 cells with/without expression of SR-B1 or LDLR by lentiviral vector, and the colonies were stained with crystal violet at 1 month post-electroporation after selection with 1 mg/mL of G418. (B) In vitro-transcribed JFH1-Nlucsec RNA was electroporated into parental, SR-KO, LD-KO and SR/LD-DKO Huh7 cells together with firefly luciferase (Fluc) RNA as an internal control, and NlucSec activity in the culture supernatants at 24, 48, 72 h post-electroporation was determined after standardization with Fluc activity. (C) Each three clones derived from parental, SR-KO, LD-KO and SR/LD-DKO Huh7 SGR cells were subjected to qRT-PCR after extraction of total RNA (upper panel) and to immunoblotting using anti-NS5A antibody (lower panel). (D) A full-length HCV RNA of JFH1 strain was electroporated into SR/LD-DKO Huh7 cells expressing SR-B1 or LDLR and the production of infectious HCV particles at 24 and 36 h post-electropolation was determined by focus forming assay. (TIF) [file ppat.1005610.s003.tif]

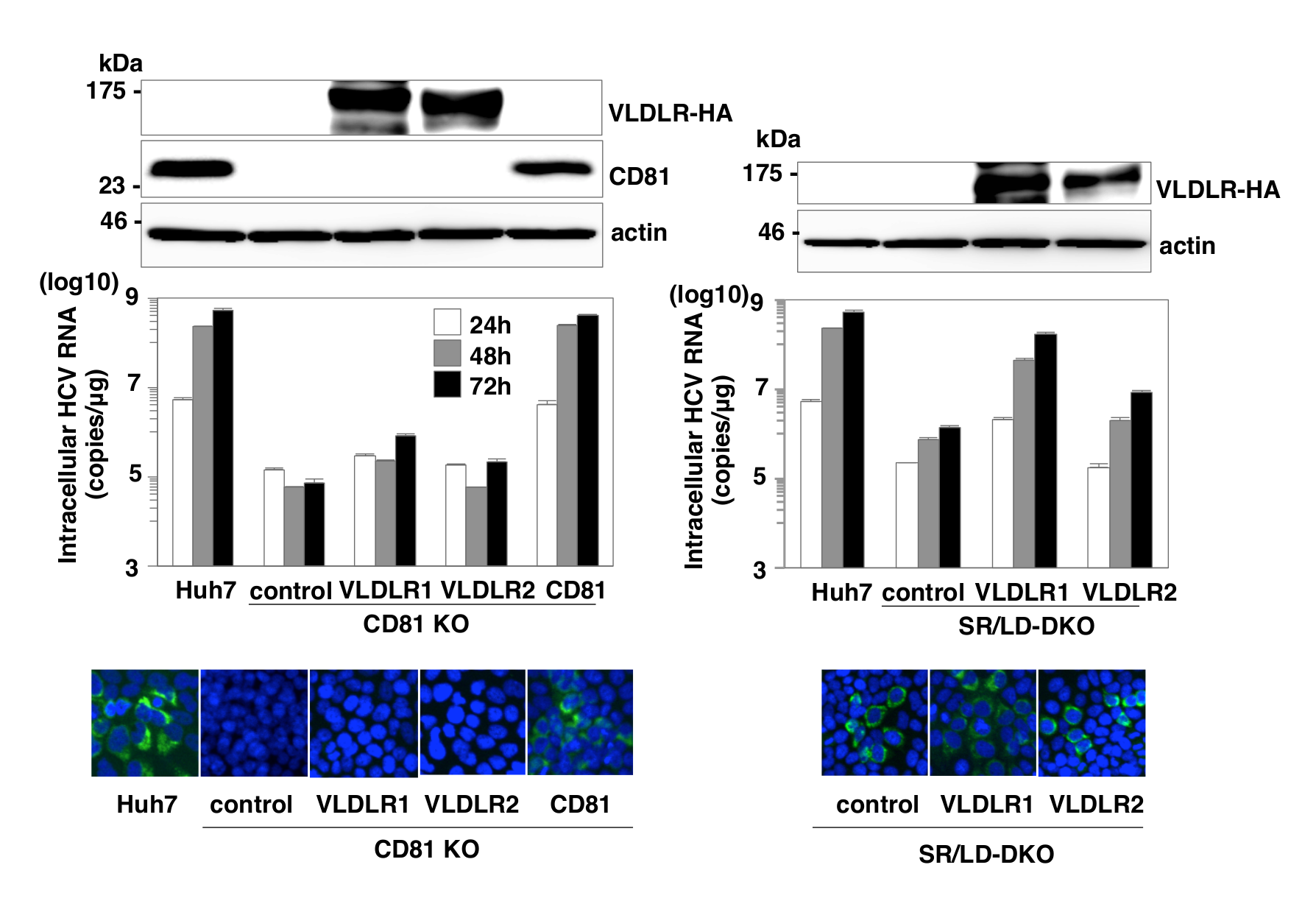

Supplement: S4 Fig — VLDLR (variant 1 or 2) or CD81 were exogenously expressed in SR/LD-DKO and CD81 KO Huh7 cells by infection with lentiviral vectors. Expressions of receptors in these cells were determined by immunoblotting analysis (upper panel). Cells were infected with HCVcc at an MOI of 1 and intracellular HCV RNA levels were determined at 24, 48, 72 h post-infection by qRT-PCR (middle panel). Cells were infected with HCVcc at an MOI of 1 and subjected to immunofluorescence analyses by using antibodies against NS5A at 72 h post-infection (lower panel). (TIF) [file ppat.1005610.s004.tif]

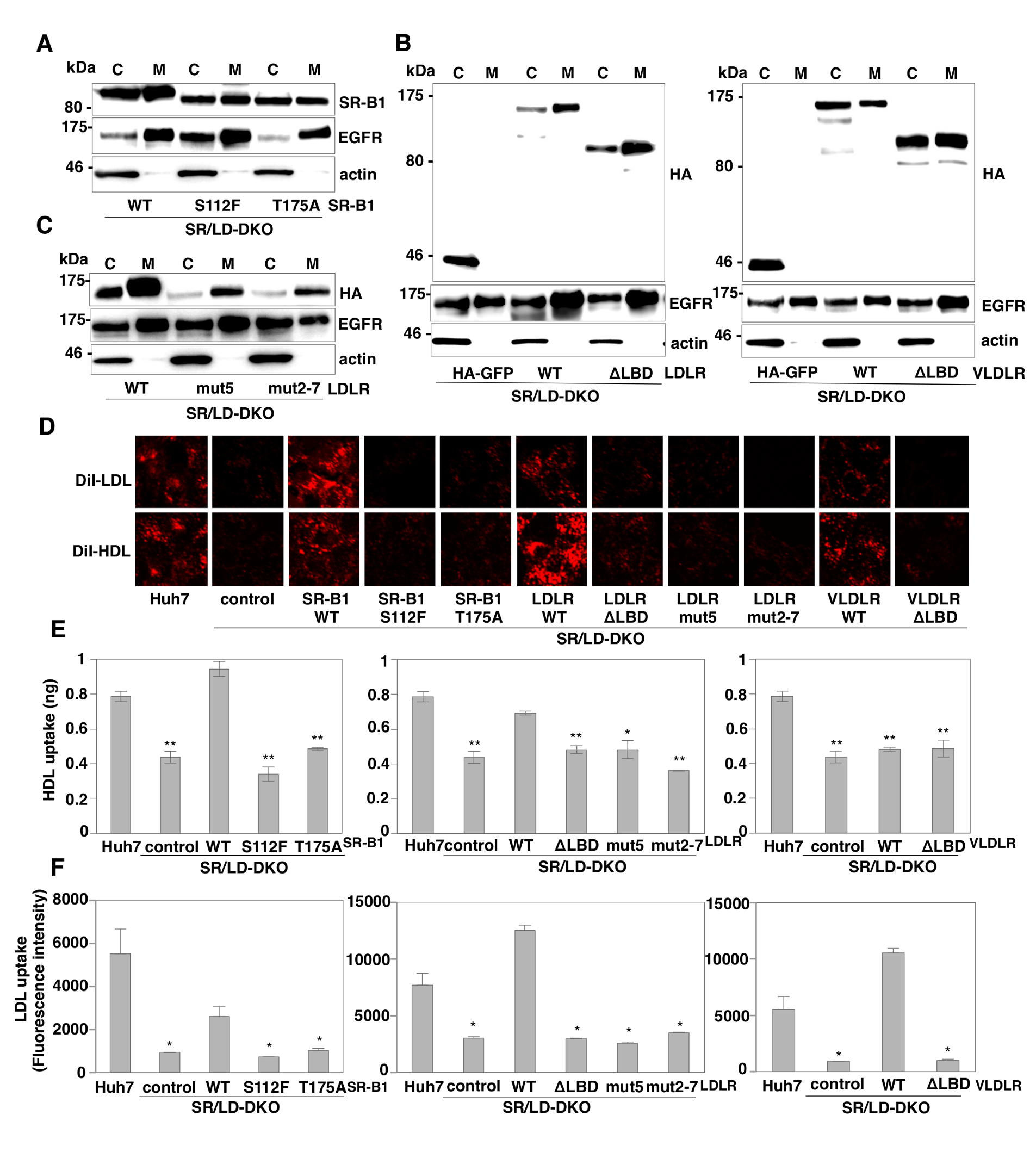

Supplement: S5 Fig — S112F- and T175A-SR-B1 missing lipid binding ability (A), ΔLBD of LDLR and VLDLR deleted the ligand binding domains (B), and mut5 and mut2-7 in which asparagine residues in the repeat 5 and in the repeats 2 to 7 of LDLR were substituted with tyrosine (C) were generated. The wild-type and these mutants of SR-B1, LDLR and VLDLR were expressed in SR/LD-DKO Huh7 cells by lentiviral vectors and cell surface proteins were biotinylated and purified by Cell Surface Protein Biotynylation and Purification Kit. Expressions of the repoprotein receptors, EGFR and actin in whole cell lysate and purified proteins were examined by immunoblotting. EGFR and actin were used as a marker of membrane and cytosolic protein, respectively. (D) Lipid uptake activities of mutants of lipoprotein receptors were determined by using lipid transfer assay. Parental Huh7 cells and SR/LD-DKO Huh7 cells expressing either wild-type or mutants of SR-B1, LDLR and VLDLR were incubated with DMEM containing 0.5% fatty acid-free bovine serum albumin and either 5μg DiI-HDL or DiI-LDL for 2h at 37°C, washed twice with PBS, and examined by a laser scanning confocal microscope. HDL (E) and LDL (F) uptake activities of parental Huh7 cells and SR/LD-DKO Huh7 cells expressing either wild-type or mutants of SR-B1 were determined by using HDL Uptake Assay Kit and PowerScanHT, respectively. In all cases, asterisks indicate significant differences (*P<0.05; **P<0.01) versus the results for Huh7 cells. (TIF) [file ppat.1005610.s005.tif]
